# Spatial transcriptomics reveals prognosis‐associated cellular heterogeneity in the papillary thyroid carcinoma microenvironment

**DOI:** 10.1002/ctm2.1594

**Published:** 2024-03-01

**Authors:** Kai Yan, Qing‐Zhi Liu, Rong‐Rong Huang, Yi‐Hua Jiang, Zhen‐Hua Bian, Si‐Jin Li, Liang Li, Fei Shen, Koichi Tsuneyama, Qing‐Ling Zhang, Zhe‐Xiong Lian, Haixia Guan, Bo Xu

**Affiliations:** ^1^ Guangdong Cardiovascular Institute Guangdong Provincial People's Hospital Guangdong Academy of Medical Sciences Guangzhou China; ^2^ Chronic Disease Laboratory Institutes for Life Sciences South China University of Technology Guangzhou China; ^3^ Guangdong Provincial Key Laboratory of Artificial Intelligence in Medical Image Analysis and Application Guangzhou China; ^4^ School of Biomedical Sciences and Engineering South China University of Technology Guangzhou International Campus Guangzhou China; ^5^ Department of Thyroid Surgery Guangzhou First People's Hospital South China University of Technology Guangzhou China; ^6^ Medical Research Institute Guangdong Provincial People's Hospital (Guangdong Academy of Medical Sciences) Southern Medical University Guangzhou China; ^7^ Department of Pathology and Laboratory Medicine Institute of Biomedical Sciences Tokushima University Graduate School Tokushima Japan; ^8^ Department of Pathology Guangdong Provincial People's Hospital (Guangdong Academy of Medical Sciences) Southern Medical University Guangzhou China; ^9^ Guangdong Provincial People's Hospital (Guangdong Academy of Medical Sciences) Southern Medical University Guangzhou China; ^10^ Department of Endocrinology Guangdong Provincial People's Hospital (Guangdong Academy of Medical Sciences) Southern Medical University Guangzhou China

**Keywords:** cellular heterogeneity, ligand‒receptor interactions, papillary thyroid carcinoma, prognosis, spatial transcriptomics, tumour microenvironment

## Abstract

**Background:**

Papillary thyroid carcinoma (PTC) is the most common malignant endocrine tumour, and its incidence and prevalence are increasing considerably. Cellular heterogeneity in the tumour microenvironment is important for PTC prognosis. Spatial transcriptomics is a powerful technique for cellular heterogeneity study.

**Methods:**

In conjunction with a clinical pathologist identification method, spatial transcriptomics was employed to characterise the spatial location and RNA profiles of PTC‐associated cells within the tissue sections. The spatial RNA‐clinical signature genes for each cell type were extracted and applied to outlining the distribution regions of specific cells on the entire section. The cellular heterogeneity of each cell type was further revealed by ContourPlot analysis, monocle analysis, trajectory analysis, ligand–receptor analysis and Gene Ontology enrichment analysis.

**Results:**

The spatial distribution region of tumour cells, typical and atypical follicular cells (FCs and AFCs) and immune cells were accurately and comprehensively identified in all five PTC tissue sections. AFCs were identified as a transitional state between FCs and tumour cells, exhibiting a higher resemblance to the latter. Three tumour foci were shared among all patients out of the 13 observed. Notably, tumour foci No. 2 displayed elevated expression levels of genes associated with lower relapse‐free survival in PTC patients. We discovered key ligand–receptor interactions, including LAMB3–ITGA2, FN1–ITGA3 and FN1–SDC4, involved in the transition of PTC cells from FCs to AFCs and eventually to tumour cells. High expression of these patterns correlated with reduced relapse‐free survival. In the tumour immune microenvironment, reduced interaction between myeloid‐derived TGFB1 and TGFBR1 in tumour focus No. 2 contributed to tumourigenesis and increased heterogeneity. The spatial RNA‐clinical analysis method developed here revealed prognosis‐associated cellular heterogeneity in the PTC microenvironment.

**Conclusions:**

The occurrence of tumour foci No. 2 and three enhanced ligand–receptor interactions in the AFC area/tumour foci reduced the relapse‐free survival of PTC patients, potentially leading to improved prognostic strategies and targeted therapies for PTC patients.

## Graphical abstract

- A new ‘RNA‐Clincal’ methodology to analyse the thyroid cancer microenvironment by combining spatial transcriptomics with clinical information identified by the pathologists.
- Atypical follicular cells (AFCs) represent a transition state from follicular cells to tumour cells.
- Tumour tissue contains heterogeneous tumour foci, and certain tumour foci are rich in genes that can predict the survival of PTC patients.
- The ligand–receptor interaction between myeloid‐derived TGFB1 and tumour foci contributed to tumourigenesis and increased heterogeneity.

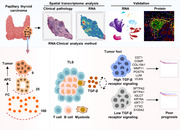

## 1 INTRODUCTION

Papillary thyroid carcinoma (PTC) is the most common malignant tumour of the endocrine system, and its incidence has been rapidly increasing in China over the past decade.^1^, ^2^, ^3^ While the prognosis for most PTC patients is favourable, a substantial proportion of patients still experience progressive invasive disease. The prognosis and therapeutics of PTC can be affected by several factors, including tumour differentiation and dedifferentiation,^4^, ^5^, ^6^, ^7^ atypical follicular cells (FCs) (AFCs) (atypical cells with focal nuclear changes, extensive but mild nuclear changes)^8^, ^9^, ^10^, ^11^, ^12^ and immune cells^13^, ^14^ in the tumour microenvironment.^15^ Therefore, it is crucial to explore the cellular heterogeneity in the PTC tumour microenvironment. Generally, the cell type and tumour area in PTC tissues are defined by a pathologist using classic pathological examination.^16^, ^17^ However, due to the variations in pathologists' expertise and insufficient spatial resolution of visual methods, pathological examinations are insufficient to fully elucidate the cellular heterogeneity of PTC, especially the discovery of valuable atypical tumour cells. Hence, methods with high precision and throughput, such as single‐cell RNA sequencing (scRNA‐seq),^6^, ^7^ transcriptomics (RNA‐seq) and proteomics analyses,^5^, ^18^, ^19^, ^20^ have been gradually applied to cell heterogeneity studies in cancers, including PTC. For example, researchers characterised the landscape underlying the initiation and progression of thyroid cancer,^4^, ^5^, ^6^, ^21^ the dedifferentiation of thyroid cancer^7^ and the immune landscape of PTC^6^, ^22^ using scRNA‐seq. They provided new insights into the understanding of PTC, but the omission of tissue in situ information limited these research efforts.

Spatial transcriptomics (SPT) has emerged as a powerful tool for investigating cellular heterogeneity based on spatial gene expression.^23^, ^24^, ^25^, ^26^ SPT has been extensively applied in the study of organ development in humans,^27^, ^28^, ^29^, ^30^, ^31^, ^32^ hematopoietic stem cells^33^, ^34^, ^35^ and various types of tumours.^36^, ^37^, ^38^, ^39^, ^40^ However, SPT has not been used to clarify the spatial cellular heterogeneity of PTC, particularly the spatial distribution of tumour cells, immune cells and normal tissue cells within or surrounding tumour foci, which significantly influence cellular functions and intercellular dynamics within tissues.^41^, ^42^, ^43^

Analytical approaches for SPT data include clustering of spatial spots based on gene expression patterns,^25^ identification of tumour areas through pathological observations and extraction of clusters using t‐distributed stochastic neighbour embedding (tSNE) for dimensionality reduction analysis.^39^ Mapping cell subpopulations from scRNA‐seq onto SPT enables the identification of accurate spatial cell populations,^29^, ^37^ and signature genes derived from scRNA‐seq clusters can be used to determine spatial cell types.^40^ However, these previous studies have primarily focused on RNA‐based analyses without integrating spatial location information. Moreover, these existing analytical methods have certain limitations, such as focusing solely on RNA information and lacking integration with clinical pathology data provided by pathologists. This may result in false positive and false negative results to some extent. To address these limitations, our research aims to combine spatial transcriptome analysis with clinicopathological diagnosis, leveraging the strengths of RNA‐based clustering and clinical pathology information.

In this study, to characterise the spatial distribution and RNA profiles of PTC‐associated cells, we collected thyroid cancer tissues from four PTC patients and used SPT in conjunction with clinical pathology identification methods. By extracting spatial RNA‐clinical signature genes for each cell type, we outlined the distribution regions of specific cells throughout the tissue sections. Various analytical approaches, including ContourPlot analysis, monocle analysis, trajectory analysis, ligand‒receptor analysis and gene set enrichment analysis, were employed to reveal the cellular heterogeneity within each cell type. Moreover, special tumour foci and three enhanced ligand‒receptor interactions within the AFC area/tumour foci were found to be associated with the prognosis of PTC patients. By integrating RNA‐based clustering with clinical pathology information, our method offers improved accuracy and comprehensive insights into spatial transcriptome analysis and prognosis‐associated cellular heterogeneity in the PTC microenvironment, which provides valuable implications for prognosis evaluation in PTC patients.

## 2 MATERIALS AND METHODS

### 2.1 Human specimens

All procedures and the use of tissue were performed according to recent national ethical guidelines. Five patients diagnosed with PTC who underwent surgery at the Second Affiliated Hospital of the South China University of Technology were included. Table S1 provides a summary of the clinical information of the patients.

### 2.2 Sample preparation and selection

Plastic embedding moulds (8 mm × 8 mm) were filled with OCT (TissueTek Sakura) at room temperature. Fresh samples were washed twice with DMEM (Gibco), and areas of interest with a base area of less than 6 mm × 6 mm were excised. The excised tissues were then embedded in OCT, frozen using dry ice or liquid nitrogen (without direct contact with liquid nitrogen) and stored at −80°C in 50 mL centrifuge tubes for subsequent spatial experiments.

Fresh frozen samples, stored for less than 3 months, were cryosectioned at a thickness of 10 μm at −15°C using a Leica cryostat. The sections were placed on highly adhesive slides (Citotest) for sample selection. The tissue sections were fixed in prechilled methanol (−20°C), reactivated with isopropanol and subjected to an improved haematoxylin and eosin (H&E) staining method. The staining procedure included haematoxylin staining for 1 min, bluing for 15 min, 80% ethanol for 2 min, eosin staining for 1 s, 80% ethanol for 2 min, 95% ethanol for 2 min (twice), absolute ethanol for 2 min (twice) and environmental protection transparent agent for 2 min (twice).

Histology images of the stained sections were captured using an Aperio Imaging and Analysis System (Leica) with a 20× objective. Valid samples were selected for further 10× Visium spatial experiments based on their pathological features.^25^, ^26^, ^31^

### 2.3 SPT sequencing

The selected frozen samples were cryosectioned and carefully placed on Visium Tissue Optimization Slides or Visium Spatial Gene Expression Slides (10x Genomics). To enhance the clarity and accuracy of histological information, mirror slice slides synchronised with the Visium spatial slides were prepared, where the front sides of the two slides represent adjacent areas. The spatial tissue sections were fixed, stained and scanned following the 10x Genomics Visium Spatial protocols (10× Genomics Document CG000241), while the synchronised mirror slices were stained using the improved H&E staining method (as described in Section 2.2). Through tissue optimisation time‐course experiments, it was determined that a permeabilisation time of 12 min was optimal for PTC samples with a thickness of 10 μm. Libraries were prepared using the Visium Spatial Gene Expression workflow (10× Genomics Document CG000239). The quality and integrity of the library DNA fragments were assessed using an Agilent 2100 instrument. The qualified libraries were then subjected to sequencing on the Illumina NovaSeq 6000 platform.^25^, ^26^, ^31^

### 2.4 SPT data processing

The raw images were exported as TIFF files using Aperio ImageScope Software (Version 12.3) with both low‐ and high‐resolution settings. The areas containing spatial barcodes were identified and marked on Loupe Browser Software (Version 6.0), and the marked areas were exported as JSON files. The Visium spatial RNA‐seq output FASTQ files, spatial barcodes and histology images were processed using Space Ranger software (Version 1.1.0) to generate feature‐spot matrices.^25^, ^26^, ^31^

For SPT data processing, the feature‐spot matrices containing histology features for each sample (as obtained from Visium raw data processing) were loaded into R (Version 4.0.5) using the Seurat R package (Version 4.0.2).^25^, ^26^, ^31^ The number of UMIs (nUMI) and genes (nGene) detected at each spot was calculated initially. Quality control (QC) was performed by filtering out genes expressed in less than three spots, mitochondrial genes and ribosomal genes. After QC, the IntergrateData function of the Seurat R package was utilised to integrate the expression data and remove batch effects (canonical correlation analysis [CCA] and RPCA, canonical correspondence analysis and reciprocal principal component analysis [PCA]) across different patients. The integrated expression matrix was then normalised, log‐transformed, centred and scaled to calculate the common variable genes. PCA of the 2000 highly variable genes was conducted to obtain a low‐dimensional space. The top five principal components (PCs) were selected as relevant dimensions for performing nearest‐neighbours‐based clustering and UMAP (uniform manifold approximation and projection) or tSNE visualisation analysis. Consensus subgroups of patients were defined based on RNA expression matrices, followed by analysis using individual sample RNA signatures combined with histological features.

### 2.5 Spatial‐geneset‐score processing

Spatial‐geneset‐score processing involved using cell‐type‐based signature genes as a gene set.^40^, ^44^ The spatial‐geneset‐score was calculated as the average log‐transformed normalisation values of the signature genes. Since the current resolution of Visium SPT cannot assign specific cell types, the spatial‐geneset‐score of each spot was used as the score for defining the corresponding cell type.

### 2.6 Cell type identification at the transcriptomic and pathological levels

The differentially expressed genes of all identity clusters were identified by the FindAllMarkers function of the Seurat R package. The top 20 markers were used to identify the preliminary cell types at the transcriptome level.^7^ Because of the limitation of current Visium SPT resolution, each spot contains approximately 3−20 cells. We developed an improved signature‐based strategy that combines the advantages of the transcriptome and histology approaches. To obtain an accurate definition of cell type at the pathological level, Prof. Liu, a clinical pathologist of PTC (KingMed Diagnostics, Guangzhou, China), was invited to distinguish and label the precise spots with pure tumour cells (enlarged/elongated/overlapped nuclei, transparent/marginalised/glassy chromatin, irregular nuclear outlines, nuclear grooves and nuclear pseudoinclusions), FCs (normal thyroid follicular epithelium cells), AFCs (slightly enlarged, overlapping, lightly stained nuclei, but no obvious nuclear grooves, nuclear inclusions and other PTC nuclear features) and immune cells (reactive hyperplastic lymphocytes).^45^ Then, ‘pure’ spots that met both transcriptomic and pathological criteria were used to screen cell type‐based signature genes of three cell types (tumour cells, FCs including AFCs, and immune cells) (Table S2). Next, the spatial‐geneset‐score of 3 cell types in each spot was calculated based on the top 20 cell‐type‐based signature genes, respectively. The spatial‐geneset‐score was calculated as the average log‐transformed normalisation values of the signature genes. Then, a cutoff value of ‘0’ for the spatial‐geneset‐score was used to identify the final cell type. If the tumour calculation score of a spot is greater than ‘0’, the spot was defined as a ‘Tumour area’; if a spot had a FC or AFC calculation score greater than ‘0’ and a tumour calculation score less than ‘0’, the spot was defined as an ‘FC area’; and if the immune calculation score of a spot was greater than ‘0’, and the tumour & FC calculation score are less than ‘0’, the spot was defined as an ‘Immune area’; and if the calculation score of all cell in a spot are less than ‘0’ the spot was defined as an ‘NA area’ (Table S3).

### 2.7 Cell trajectory analysis

The monocle developmental trajectory was calculated on monocle 3 (the method used by SPATA2^46^) or monocle 2 (monocle package, version 2.18.0).^7^, ^46^, ^47^ Raw count served as input for Monocle, and pseudo‐time was computed and graphed using the ‘reduceDimension (DDRTree)’, ‘orderCells’ and ‘plot_cell_trajectory’ functions. Trajectory patterns were determined by the ‘differentialGeneTest’ function.

### 2.8 Tumour foci heterogeneity measurement

To identify the heterogeneity of tumour foci, the Seurat object was transformed to SPATA data by the SPATA2 R package (version 4.0.2).^48^, ^49^ Tumour foci were the basic subunit of the tumour, with at least three gathered tumour spots. These foci were far away from other gathered tumour foci (at least 25 μm), and the other scattered spots were also treated as tumour foci for further analysis. Tumour foci were segmented in the SPATA2 environment, with the focus of the tumour foci heterogeneity on the common subpopulations among different patients. For this reason, we used the cell label transfer method (find anchors between a reference and query object, and use the anchors to transfer data from the reference to query object^50^, ^51^) to ensure that the Visium spatial samples shared the same subtypes of tumour area, FC area and immune area. We used patient 1 to explore the heterogeneity among different tumour foci because the tumour foci of patient 1 were highly heterogeneous. Then, the tumour foci were chosen to obtain differentially expressed genes, and the monocle developmental trajectory was calculated on monocle.^7^, ^46^, ^47^ We then performed Gene Ontology (GO)/Kyoto Encyclopedia of Genes and Genomes pathway analysis and Gene Set Variation Analysis to identify the enriched key gene sets among the selected tumour foci, according to the enrichment score of gene sets from the Molecular Signatures Database. Survival analysis of signature genes on PTC were done on the Gepia2 database (based on the thyroid carcinoma data of The Cancer Genome Atlas [TCGA] Program).^52^

### 2.9 External tumour microenvironment cell distribution measurement

The distances between cells and tumour foci were calculated to identify the cell distribution of the tumour microenvironment. The cell position information was extracted. The distance between selected cells and all tumour cells was calculated, and the shortest distance was used to classify cells surrounding the tumour foci (Get‐nearby‐cell function). One‐way analysis of variance (ANOVA) was performed for the fractions of cell types surrounding all tumour foci to obtain the cell distribution in the external tumour microenvironment. Next, the surrounding cell features and the cluster interaction of tumour and microenvironment cells were identified through cluster trajectory analysis. In the SPATA2 environment, the cell trajectory from tumour to FCs passing by AFCs was drawn, and the biological features (genes or gene sets) were fitted according to the internal standard mathematical model (including linear ascending/descending, logarithmic ascending/descending, sinus and sinus (reversed)).^48^, ^49^

### 2.10 Contour map data processing

For each biological feature (genes or geneset‐based score), we generated a contour map to show the value distribution on the spatial transcriptome plane.^53^, ^54^ We used the Python package Matplotlib to draw the contour map, which requires a 2‐D numeric matrix as the input. The 10× Visium array arranges the data spots in a discontinuous style on the 2‐D rectangular coordinate system. For a column with an odd/even *X* coordinate, there are only odd/even values for *Y*. Therefore, the actual data spots cover only half of the spots within the tissue region. For one biological feature, we filled the discontinuous spots within the tissue region with the mean value of 4 adjacent spots with actual data, and all the spots outside the tissue region were filled with the minimum value minus the standard deviation of the values of actual data spots. A contour map contains many contour line regions at several levels of the biological feature. To understand the connection between the level of biological features and the regions formed by one type of cell, we compared the overlap relationship between each contour line region and each cell type region. We defined a cell type region as the convex hull of a set of actual data spots with the same cell type. The spot set should have at least five spots with Chebyshev distances equal to or less than 2. This process was performed using the Python package NetworkX. We defined the match quality of a contour line region with a cell type region as the square of the overlap area divided by the product of the two region areas. For each cell type region, we sorted the overlapping contour line regions in descending order of the contour level and the match quality. The top genes of the region‐matched results were considered specific to the cell type region.

### 2.11 Copy number variation analysis

The data extracted from SPT data were analysed with the infercnv package (version 1.3.3) for copy number variations (CNVs).^55^, ^56^ In the infercnv analysis, we configured the parameters by setting ‘denoise’, ‘default hidden Markov model (HMM)’ and ‘cluster_by_groups’ to TRUE. To analyse the gene CNV expression changes at various locations on the genome of tumour cell and AFC cells, the FCs were used as the reference cell, and its modified CNV expression value was defined as 1. Hence, the modified CNV level (relative CNV expression) of genes on each chromosome were summarised in the heatmap. These genes with modified CNV level greater than or less than 1 imply more gain or loss. And the top gain and loss genes were then extracted based on the modified CNV value and showed in the violin plots. On the other hand, we also evaluated and graphed the CNV values of some genes that are commonly genetic altered in PTC disease, including BRAF, RAS, AKT, PTEN, PIK3CA mutations, as well as PAX8–PPARG fusions. We calculated the CNV values of these genes and compared their values between AFCs and tumour cells. Gain of an oncogene may also represent an oncogenic stimulus, while loss can substitute for inactivating mutations of tumour suppressors.^57^ Furthermore, the correlation of sample mutations in FCs, AFCs and tumour cells were calculated based on the relative expression of all genes.

### 2.12 Single‐cell RNA sequencing data analysis

The data extracted from GSE184362, GSE158291 and GSE232237 were reanalysed with the Seurat package.^4^, ^22^ Specifically, cells from PTC patients were filtered based on the label in the meta data; cells with percent.mt < 40%, UMIs > 200 and UMIs < 6000 were filtered; and the top 2000 VariableFeatures were used for downstream data scale, ‘RunPCA’, ‘FindNeighbors’, unsupervised clustering and dimensional reduction analysis. The entire thyrocyte population (tumour cells and FCs) and immune populations (T, NK, B and myeloid) were isolated for further analysis. Then, we employed the cell label transfer function to identify anchors between a reference patient and query patients and then transferred the cell label data. Patient No. 1 in our work was selected as the reference patient, and the patients from scRNA‐seq data (GSE184362, GSE158291 and GSE232237) were served as query patients, respectively. This analysis enabled the identification of both common and unique clusters of AFCs, FCs and tumour cells in the entire thyrocyte population of these scRNA‐seq data (GSE184362, GSE158291 and GSE232237). Besides, the expression patterns of TGFB1 and CXCL13 in the scRNA‐seq data (GSE184362) were analysed. The composition and percentage of clusters were further identified and calculated in R.

### 2.13 Multiplex immunohistochemistry

FFPE tissue sections were stained with the PANO 7‐plex IHC kit (Panovue) according to the manufacturer's instructions, including deparaffinisation with xylene, hydration with gradient ethanol, antigen retrieval with sodium citrate buffer (0.01 M, pH = 6.0), removal of endogenous peroxidase with H_2_O_2_, blocking tissues with 10% goat serum and staining with antibodies and TSA‐RM. The next cycle of staining started with antigen retrieval and was stopped with TSA labelling. The antibodies used included CD4 (ab133616, 1:500; Abcam) with Opal 620, CD8 (C8/144B, 1:200; CST) with Opal 690, and CD20 (ab78237, 1:2000; Abcam) with Opal 520. The antibodies used included CD45 (20103‐1‐AP, 1:2000; Proteintech) with Opal 570, SFRP4 (15328‐1‐AP, 1:200; Proteintech) with Opal 620, and IgG (EPR4421, 1:500; Abcam) with Opal 520. Then, all slides were scanned and analysed using the Vectra Automated Quantitative Pathology Imaging System (Vectra Polaris featuring MOTiF™). Finally, the percentage of SFRP4^+^ AFCs around the Tumour area was analysed in the HALO software (version 3.3). Specifically, the typical tumour area were identified based on DAPI staining, the distance and the area of 0–2, 2–4 and >4 mm from the tumour area were delineated, and the percentage of SFRP4^+^ AFCs in DAPI^+^ cells were calculated and statistical analysed.

### 2.14 Statistical analysis

All statistical data are presented as the mean ± standard deviation (SD). The normality test and homogeneity of variance test were conducted for unpaired samples (*n* ≥ 3) (GraphPad Prism, Version 9.0). For two sets of samples, if normality and variance homogeneity were met, a two‐tailed unpaired Student's *t*‐test was used; if not, the Mann–Whitney test was used. For multiple sets of samples, one‐way ANOVA was used if the sets were normally distributed; otherwise, a nonparametric test was used. In the scRNA‐seq data and spatial transcriptomes data, the difference of gene expression across the populations were calculated by wilcox.test function in R (Mann–Whitney’ test). A *p* value < .05 was considered statistically significant (*p < .05; **p < .01; ***p < .001).

## 3 RESULTS

### 3.1 Establishment of a spatial RNA‐clinical analysis method for the PTC microenvironment

**FIGURE 1:**
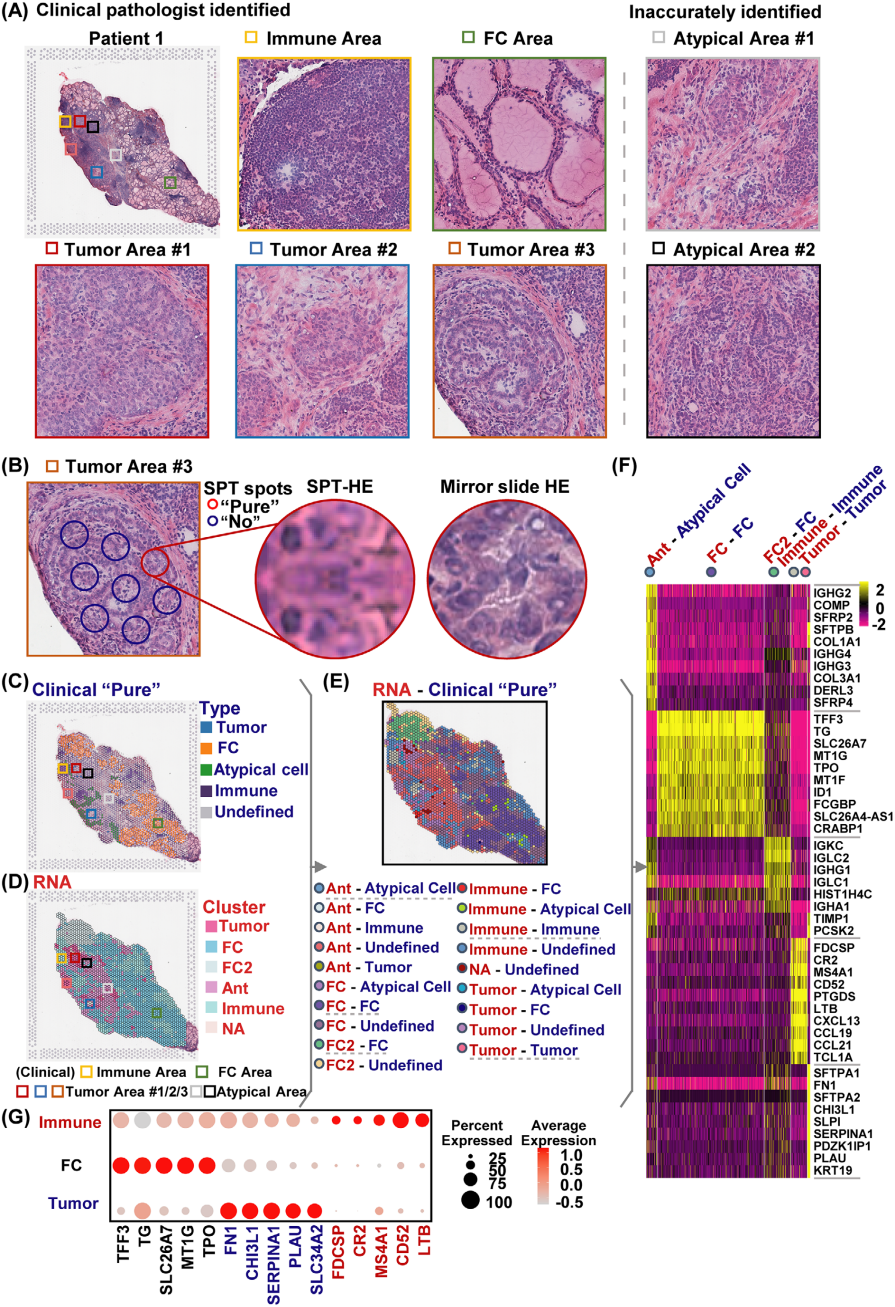
A novel cluster definition method integrating spatial transcriptomics with clinical pathology data. (A) Improved haematoxylin and eosin (H&E) staining results and magnified images of selected areas on a mirror slide of spatial transcriptomics from papillary thyroid carcinoma (PTC) samples. The clinical pathologist accurately identified the typical immune area (yellow box), FC area (green box) and tumour areas (red, blue and orange boxes). Inaccurately identified atypical areas are represented by grey and black boxes. (B) The clinical pathologist accurately identified the ‘pure’ tumour spot (red cycle) and ‘not pure’ tumour spots (blue cycle) in the typical tumour area (orange boxes). Magnification images of improved H&E staining (HE) and spatial HE staining (SPT‐HE) results on selective ‘pure’ tumour spot of PTC. (C) The corresponding clinical ‘pure’ type of PTC identified by the clinical pathologist is shown. (D) Spatial mRNA identification of clusters in patient No. 1 of PTC using spatial transcriptomics analysis. (E) The merged analysis image displays the RNA clusters (indicated by red letters) and clinical types (indicated by blue letters). The grey dashed line represents the clusters which meet the same RNA clusters and clinical types, including Ant, atypical cell; FC, FC; FC2, FC; immune, immune cell; tumour, tumour cell. (F) Heatmap of the typical RNA‐clinical cluster top 10 genes, including Ant, atypical cell (blue); FC, FC (purple); FC2, FC (green); immune, immune cell (grey); tumour, tumour cell (pink). (G) Dot plot illustrating the expression of characteristic genes across all spatial transcriptomics samples of PTC. The characteristic genes were derived from the tumour, FCs and immune areas.

The tumour microenvironment of PTC consists of various cell types, including typical and atypical thyroid follicular cells (FCs and AFCs), tumour cells and immune cells. These cells aggregate within and around tumour foci, and their functional and distributional heterogeneity is greatly influenced by the disease state. Clinical pathologists usually accurately identify regions of FC, tumour cells and typical immune cells in intraoperative frozen PTC sections (Figure 1A, left). However, the presence of spatially atypical regions (Figure 1A, right) can lead to a certain rate of missed diagnoses, impeding the early detection and timely treatment of PTC patients.

In this study, we developed a novel spatial RNA‐clinical combination analysis method to investigate the cellular heterogeneity of PTC in greater detail. By integrating SPT data with pathological diagnostic information, this method enables more accurate extraction of signature genes and delineation of regions containing typical and AFCs, tumour cells and immune cells within PTC. To overcome the pixel limitation associated with SPT‐HE staining, we enhanced the HE staining method on a mirror slide of spatial slides (Figures 1B and S1A–S1D). The magnified HE view of the mirror slides exhibited improved clarity in identifying the ‘pure’ FC spot, tumour spot and immune spot compared with SPT‐HE staining (Figures 1B and S1A–S1D). Concurrently, clinical pathologists identified the clinically ‘pure’ tumour cells, FCs, AFCs and immune cells (Figures 1C and S1E).

The SPT data from tissue sections belonging to four PTC patients were merged using CCA (Figure S2A). Subsequently, 14 common clusters were identified based on highly variable genes and top principal components, as depicted in the UMAP plot (Figure S2B) and spatial distribution plot (Figure S2C–S2D). The signature genes associated with these common clusters were extracted and utilised to categorise RNA clusters into five distinct cell types, namely, tumour, FC (normal follicular cells), FC2 (follicular cells with specific genes), Ant (adjacent noncancerous tissue, including AFCs, fibroblasts and blood vessel cells) and immune cells (Figures 1D and S2E). By intersecting the RNA clusters with the clinically determined ‘pure’ cell types, new RNA‐clinical populations were established (Figures 1E and S2F). Cells that exhibited the same RNA clusters as the clinically ‘pure’ types were utilised to extract signature genes specific to tumour cells, immune cells, FCs (FC2) and AFCs (Figure 1F). Employing this methodology, we screened these cells across all four tissue sections (Figure S2G) and identified the top 20 signature genes for each cell type, which were defined as the spatial RNA‐clinical signature genes (Table S2). The expression patterns of the top five signature genes among the 20 identified RNA‐clinical signature genes exhibited distinct differences between tumour cells, FCs and immune cells, as illustrated in the dot plot of the merged samples from CCA analysis (Figure 1G). This observation underscores the accuracy and specificity of the spatial RNA‐clinical combination analysis method in characterising the cellular heterogeneity within PTC.

### 3.2 Application of the RNA‐clinical analysis method to the cell cluster pattern study of PTC

**FIGURE 2:**
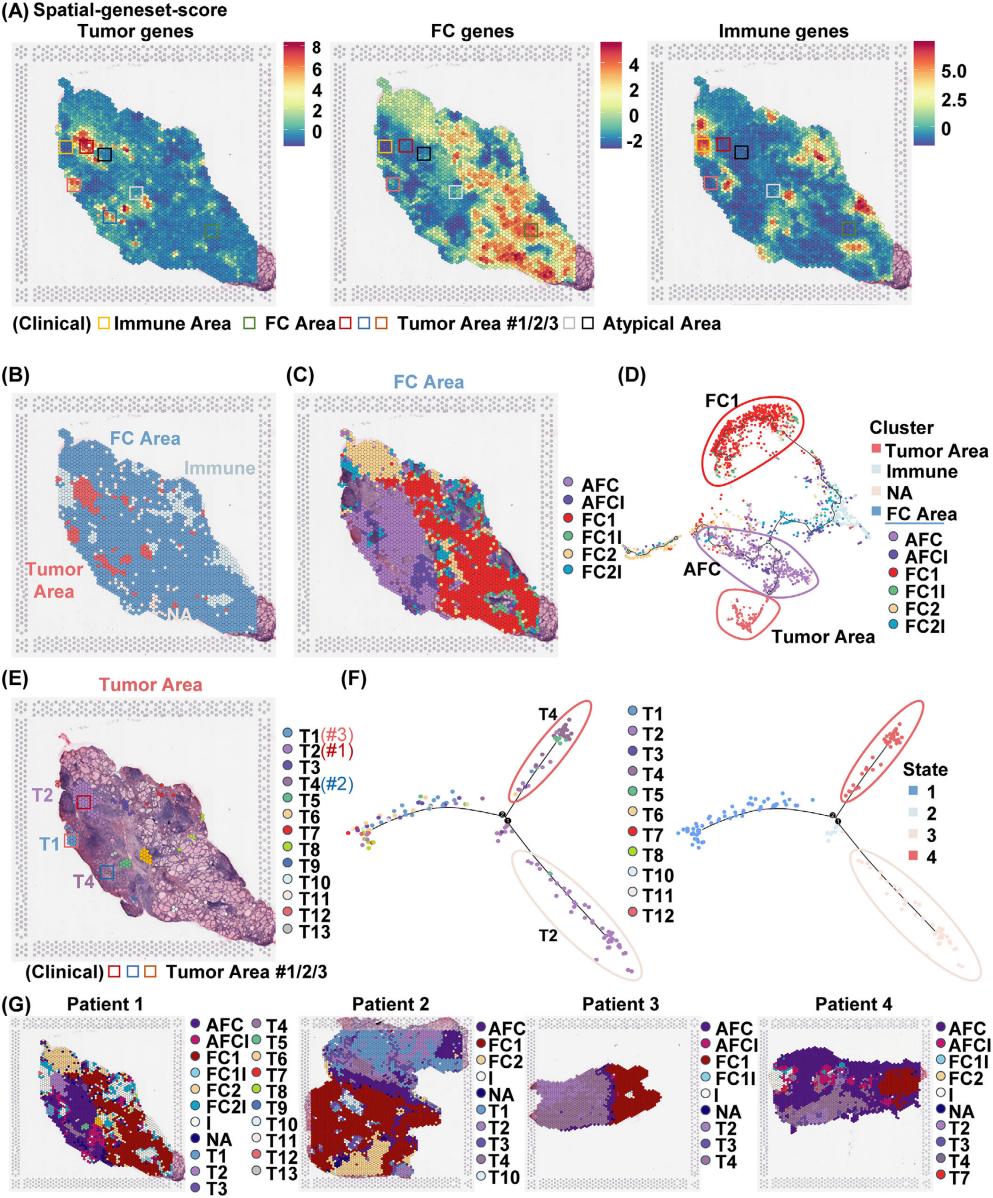
Cluster analysis of papillary thyroid carcinoma using the RNA‐clinical method. (A) Expression patterns of tumour markers, FC markers and immune markers in tissue sections. The box represents the area (immune area, FC area, tumour area and atypical area) recognised by the clinical pathologists in patient No. 1. (B) Cluster definition plot of the tumour area, FC area, immune area and nonassigned (NA) area in patient No. 1, obtained through the integration of spatial transcriptomics with mirror H&E data. (C) Subset clusters within the FC area in tissue sections, including AFCs (atypical FCs), AFCIs (atypical FCs with immune infiltration), FC1, FC1I, FC2 and FC2I. (D) Monocle plot displaying the FC area (including its subpopulations AFCs, AFCIs, FC1, FC1I, FC2 and FC2I), tumour area, immune and NA along the trajectory. The dashed circle in the monocle plot represents the typical subsets, including Tumour area (pink), AFCs (purple) and FC1 (red). (E) Thirteen tumour foci identified in tissue sections. The typical tumour areas recognised by the clinical pathologists were represented by red (tumour area #1), blue (tumour area #2) and orange boxes (tumour area #3). (F) Cell trajectory plot of tumour foci clusters and states based on monocle analysis, limited to tumour foci with at least three cells. The dashed circle in the monocle plot represents the typical subsets, including tumour foci No. 2 (pink) and tumour foci No. 4 (red). (G) The spatial dimplot of patient No. 1 (left) and the cell label transfer plots of the cell labels transferred from patient No. 1 to patients Nos. 2, 3 and 4 (right).

Using the spatial RNA‐clinical analysis method developed above, we obtained the characteristic spatial RNA‐clinical signature genes for tumour cells, FCs, AFCs and immune cells in PTC. These top 20 signature genes were then utilised to assess the spatial‐geneset‐score of each spot in the spatial transcriptome data using the previously described spatial grading system.^40^ The resulting spatial feature plot depicted the regional distribution spatial‐geneset‐score of different cell types (tumour cells, FCs and immune cells) throughout the entire tissue section (Figure 2A). In addition, our RNA‐clinical method was confirmed to be consistent with the results of the single‐cell transcriptome in some studies^21^ based on the characteristic gene calculations, suggesting that our RNA‐clinical method is accurate and specific (Figures 2A and S3A and B). The colour transition from blue to red indicates higher spatial‐geneset‐scores and the aggregation of specific cell types. The critical value for the cell spatial‐geneset‐score was defined as 0 (Table S3). Since spots in the SPT data are not directly interpreted as cells,^58^ the rules were applied to accurately identify the regions of cell clusters (Table S3). If the spatial‐geneset‐score of a spot in the tumour area exceeded the critical value, it was marked as ‘T’; otherwise, it was marked as ‘nT’ (indicating no tumour). Similarly, spots in different cell areas were compared with the critical value and labelled ‘FC’/’nFC’, ‘AFC’/’nAFC’ and ‘I’/’nI’ to represent FCs, AFCs and immune cells, respectively. The final cell label depended on the presence of ‘T’ or a combination analysis result of ‘FC’, ‘AFC’ and ‘I’ (refer to Section 2 for details). Consequently, the FC area (including typical and AFCs), tumour area, immune area and nonassigned (NA) area in PTC tissue sections were comprehensively and accurately identified (Figure 2B).

By employing this method, we not only accurately identified the cell types defined by the pathologist (Figures 1A and 2A) but also screened all distribution regions of a particular cell type within the entire tissue section, facilitating a comprehensive analysis of the cellular heterogeneity among tumour cells, FCs, AFCs and immune cells. After defining the regions of tumour cells, FCs and immune cells, the overall distribution of these cells was examined (Figure 2B). The entire FC area consisted of the following cell clusters: AFC, AFCI, FC1, FC1I, FC2 and FC2I (Figure 2C). Monocle analysis revealed that immune cells and most typical FC cells were different from the tumour cells in the developmental state, while a portion of the FCs (predominantly AFCs) was closer to the tumour cells in the developmental state (Figures 2D and S3C). The simplified pseudotime plot further demonstrated that AFCs were the cell type closest to the tumour area, indicating that AFCs might serve as transition cells between FCs and tumour cells, potentially participating in the dedifferentiation of FCs into tumour cells (Figure S3D). Furthermore, 13 tumour foci were identified, grouped and distributed within the tumour area (Figure 2E). Among them, tumour foci No. 1 (orange box), tumour foci No. 2 (red box) and tumour foci No. 4 (blue box) were also identified by clinical pathologists (Figure 2E). Cell trajectory analysis further revealed that tumour foci No. 2 and No. 4 developed in different states, as determined by monocle analysis (Figure 2F). These findings provide insights into the cell heterogeneity of AFCs and different tumour foci in PTC. Moreover, the impact of immune cells on tumour heterogeneity will be further described below. To further investigate the characteristics of cell heterogeneity, we employed the cell label transfer function to identify anchors between a reference patient and query patients and then transferred the cell label data. Patient No. 1 was selected as the reference patient, and the other patients served as query patients. This analysis enabled the identification of tumour foci across all PTC tissue sections. The spatial dimplot revealed both common and unique clusters of AFCs, FCs, immune cells and tumour foci among the tissue sections of the four patients (Figure 2G), but little is known about the tissue location and characteristic genes of AFCs and tumour foci.^8^, ^9^, ^10^, ^11^, ^12^

### 3.3 AFC represents a transition state from FCs to tumour cells

**FIGURE 3:**
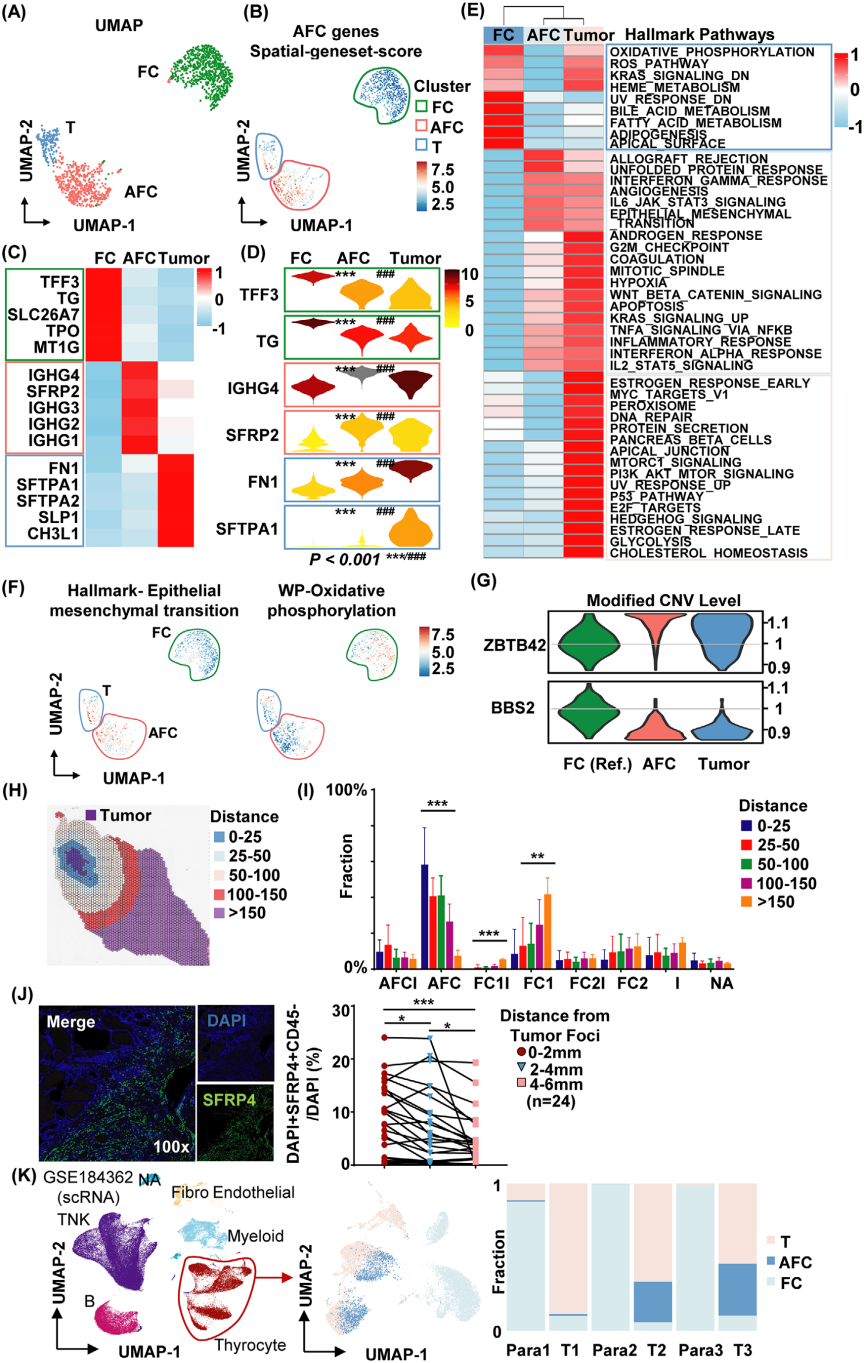
The atypical FCs were transition cells from FCs to tumour cells. (A) UMAP plot showing the intersection of tumour cells, FCs and AFCs in tissue sections. (B) Feature plot depicting the expression of AFC signature genes on tumour cells, AFCs and FCs using the spatial‐geneset‐score method. (C) Heatmap plot displaying the expression of the top five signature genes from tumour cells, AFCs and FCs. The difference of genes expression of FCs/AFCs, AFCs/tumour and FCs/tumour were calculated and showed. *^/#/&^ represents the *p* value of FC versus AFC, AFC versus tumour, FC versus tumour, respectively. ***^/###/&&&^ represents *p* value < .001. (D) Violin plot illustrating the expression of top two signature genes across tumour cells, AFCs and FCs. (E) Heatmap depicting the expression levels of total hallmark pathways in tumour cells, AFCs and FCs. (F) Feature plot depicting the expression of the ‘hallmark‐epithelium mesenchymal transition’ and ‘WP‐oxidative phosphorylation’ gene sets in tumour cells, AFCs and FCs. (G) Violin plot of modified CNV levels of the top gain (ZBTB42) and loss (BBS2) mutation genes across FCs, AFCs and tumour cells. FCs were used as the reference cells. (H) External segmented distribution based on the nearest distances to tumour foci No. 2. (I) Bar chart presenting the statistical analysis of the cell fraction of the tumour foci external microenvironment grouped by distances. (J) Multiplex immunohistochemical staining image (left) and statistical analysis results (right) of the distribution of AFCs (SFRP4‐positive) in tissue sections around tumour foci by distances. *p < .05; **p < .01; ***p < .001. (K) UMAP plot illustrating the distribution of clusters in the single‐cell sequencing data (left, GSE184362) and bar plot depicting the distribution of tumour cells, AFCs and FCs across the PTC patients (right).

As shown above, the pseudotime plot revealed that AFCs exhibited closer proximity to tumour cells than FCs (Figure S3D). To gain a deeper understanding of the cell heterogeneity within AFCs and its relationship with FCs and tumour cells in PTC, we constructed UMAP plots and spatial‐geneset‐score plot to visualise the developmental trajectories of AFCs, FCs and tumour cells (Figure 3A). The UMAP plot illustrated a closer relationship between AFCs and tumour cells compared with FCs (Figure 3A). Moreover, the spatial‐geneset‐score plot of signature genes demonstrated that the expression pattern of certain AFCs resembled that of tumour cells (Figures 3B and S4A). Furthermore, we examined the gene expression levels of AFCs and tumour cells and observed similarities in the expression patterns of top five signature genes (Figure 3C). Notable genes displaying similar expression patterns in AFCs and tumour cells compared with FCs included TFF3 and TG (signature genes of FCs), IGHG4 and SFRP2 (signature genes of FCs) and FN1 and SFTPA1 (signature genes of tumour cells) (Figures 3C and D). Especially, the immunoglobin gene IGHG4 was highly expressed in the AFC area (Figure S4B). GO term enrichment analysis demonstrated a greater overlap in up‐regulated GO pathways between tumour cells and AFCs than between tumour cells and FCs (Figure S4C). Similar trends were observed in the spatial‐geneset‐score analysis results of hallmark gene pathways in FCs, AFCs and tumour cells (Figure 3E). For instance, enrichment of the epithelial–mesenchymal transition (EMT) pathway was observed in AFCs and tumour cells but not in FCs, while the oxidative phosphorylation pathway exhibited higher enrichment in FCs than in AFCs and tumour cells (Figure 3F). Moreover, CNV^56^ analysis of the key genes in PTC (such as KRAS, AKT1 and PIK3CA)^55^ and the top gain and loss genes, and analysis of whole chromosome genes were conducted in FCs, AFCs and tumour cells (Figures 3G and S4D–S4F). The clustering results of cells (Figure S4D), the modified CNVs expression pattern of genes (Figure 3G, S4E) and the correlation analysis result of CNVs (Figure S4F) further supported the notion that AFCs represent a transition state that is more closely related to tumour cells. These findings indicate that AFCs represent a transition state from FCs to tumour cells.

Additionally, we evaluated the cellular composition of the external tumour microenvironment based on the distance from the tumour foci (Figures 3H, I and S4G, S4H). The analysis revealed a decrease in the number of AFCs and an increase in the number of FCs as the distance from the tumour foci increased in patient No. 1 (Figure 3I). The number of AFCs decreased across all patients (Figure S4H). We also conducted a multiplex immunohistochemical staining experiment on PTC tumour tissue and found that the number of AFCs decreased as the distance from the tumour foci increased (Figures 3J and S4I). Importantly, evidence from online single‐cell RNA‐seq data (GSE184362, GSE158291 and GSE232237)^4^, ^22^ also indicated the existence of AFCs and the similarity between tumour cells and AFCs compared with FCs (Figures 3K and S4J, S4K). Collectively, our work provides additional evidence highlighting the heterogeneity and transition state of AFCs from FCs to tumour cells.

### 3.4 The key ligand–receptors interactions between AFCs and tumour foci

To explore the effects of AFCs on tumour foci, we conducted an analysis of cell‒cell communication involving tumour cells, AFCs and FCs based on ligand‒receptor pathways in the PTC tumour microenvironment. Cell‒cell communication, particularly through ligand‒receptor pathways, plays a crucial role in modulating cellular functions and distribution within the tumour microenvironment. We observed that AFCs were enriched around tumour foci (Figures 3H–J). Initially, we manually drew a trajectory line from tumour cells and AFCs to FCs in the PTC SPT data using the SPATA2 package (Figures S5A–C). Ligand‒receptor pathway analysis revealed a decreasing intensity trend of the FN1–SDC4 and FN1–ITGA3 pathways along this trajectory (Figure S5C). However, manually drawn trajectory lines are limited by the contingency of trend line location and orientation and a one‐sided perspective based on the trajectory direction rather than the entire tumour foci. To overcome these limitations, we introduced the concept of contour lines from mathematics and geography. Specifically, we projected the expression pattern of the FN1 feature plot into a ContourPlot, demonstrating high similarity and feasibility (Figure S5D). Subsequently, ligand‒receptor pairs were evaluated and projected into a contour plot using the contour plot analysis method. The top three ligand‒receptor pathways from tumour cells to AFCs to FCs were identified as FN1–SDC4, FN1–ITGA3 and LAMB3–ITGA2 (Figures S5E and F). Notably, high expression levels of FN1–SDC4, FN1–ITGA3 and LAMB3–ITGA2 were associated with a decreased relapse‐free survival fraction of PTC based on gene expression profile analysis from TCGA database (Figure S5G). This suggests that FN1–SDC4, FN1–ITGA3 and LAMB3–ITGA2 may function as potential prognostic biomarkers for PTC. Overall, our analysis provides insights into the functions of AFCs and their role in cell‒cell communication within the PTC tumour microenvironment. The findings highlight the transition state of AFCs from FCs to tumour cells and their potential impact on the progression and heterogeneity of PTC. Additionally, the identified ligand‒receptor pathways involving AFCs may have implications for the prognosis and therapeutic targeting of PTC.

### 3.5 The heterogeneity of tumour foci in PTC

**FIGURE 4:**
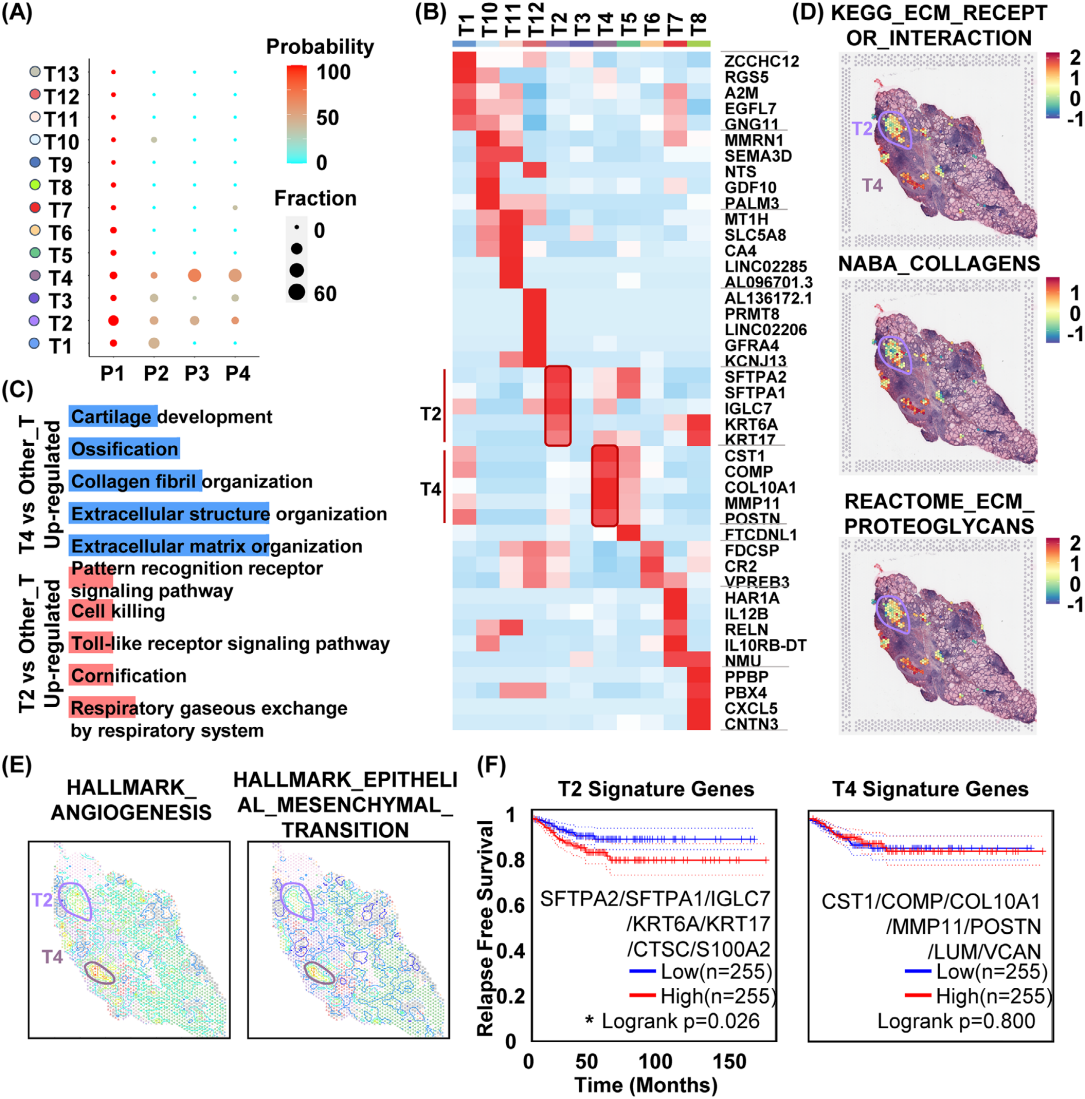
Heterogeneity of tumour foci in PTC. (A) Statistical dot plot illustrating the distribution of tumour foci among all tissue sections from patients Nos. 1−4. (B) Heatmap presenting the expression levels of the top five signature genes in total tumour foci of patient No. 1, excluding tumour foci with fewer than three cells. (C) Up‐regulated GO pathways in tumour foci No. 2 or tumour foci No. 4 compared with other tumour foci. (D) SpatialFeaturePlot displaying the spatial‐geneset‐score of ECM‐receptor‐or‐interaction (Kegg), collagens (Naba) and ECM‐proteoglycans (Reactome) on tumour foci No. 2 and tumour foci No. 4. (E) SpatialFeaturePlot illustrating the spatial‐geneset‐score of metastasis‐EMT‐UP (Alonso) and metastasis‐UP (Chandran) on tumour foci No. 2 and tumour foci No. 4. (F) Relapse‐free survival plot of thyroid carcinoma (THCA, TCGA database) based on the top seven signature genes derived from tumour foci No. 2 or tumour foci No. 4.

Besides the heterogeneity of FC area (including AFCs and FC), we also noted heterogeneity of tumour foci in the pseudotime plot analysis of tumour cells (Figure 2F) and clinical definition of tumour area (Figure 1A). The statistical analysis of all tumour foci in the four samples demonstrated that tumour foci Nos. 2, 3 and 4 were shared among all PTC patients, while tumour foci Nos. 1 and 10 were shared by two patients. Other tumour foci were observed in only one patient (Figure 4A). Therefore, our focus was primarily on tumour foci Nos. 2, 3 and 4. Among these, tumour foci No. 2 exhibited high expression of surfactant protein A2 (SFTPA2), surfactant protein A1 (SFTPA1), immunoglobulin lambda constant 7 (IGLC7), keratin 6A (KRT6A) and keratin 17 (KRT17) genes, whereas tumour foci No. 4 demonstrated high expression of cystatin SN (CST1), cartilage oligomeric matrix protein (COMP), collagen type 10 alpha 1 chain (COL10A1), matrix metallopeptidase 11 (MMP11) and periostin (POSTN) genes. Tumour foci No. 3 did not exhibit prominent expression of specific genes (Figure 4B).

GO enrichment analysis revealed that pathways such as ‘respiratory gaseous exchange by respiratory system’, ‘cornification’, ‘Toll‐like receptor (TLR) signalling pathway’, ‘cell killing’ and ‘pattern recognition receptor signalling pathway’ were up‐regulated in tumour cells from tumour foci No. 2 (Figure 4C). TLR signalling is known to promote tumour growth, while cornification represents a unique form of terminal differentiation and programmed cell death. However, in tumour cells from tumour foci No. 4, pathways related to ‘extracellular matrix (ECM) organisation’, ‘extracellular structure organisation’, ‘collagen fibril organisation’, ‘ossification’ and ‘cartilage development’ were up‐regulated (Figure 4C). ECM effects and collagen formation play important roles in tumour cell metastasis. Spatial‐geneset‐score analysis of these pathways confirmed the enrichment of ‘kegg ECM_receptor_interaction’, ‘naba collagens’ and ‘reactome ECM_proteoglycans’ in tumour foci No. 4 (Figure 4D). Additionally, analysis using ContourPlot revealed that hallmark angiogenesis and EMT pathways were more enriched in tumour foci No. 4 than in No. 2, suggesting a potential facilitation of tumour cell metastasis (Figure 4E). Notably, tumour foci No. 2 exhibited a similar but different level of CNV across the entire genomic region compared with tumour foci No. 4 (Figures S6A–S6C). Moreover, high expression of the top seven genes from tumour foci No. 2 was associated with decreased relapse‐free survival in patients (Figure 4F).

### 3.6 Tumour immune microenvironment affected tumour foci heterogeneity in PTC

**FIGURE 5:**
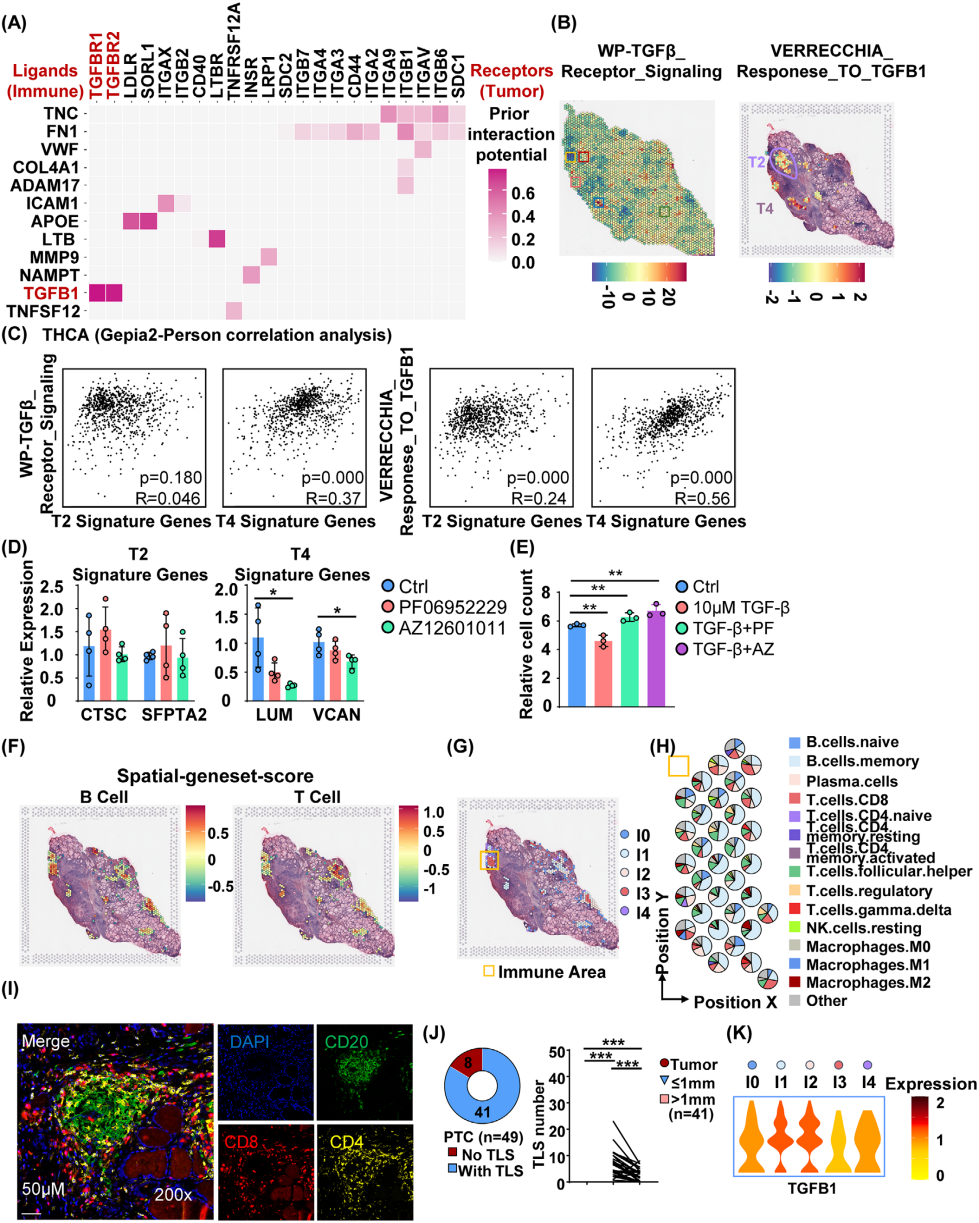
The interaction between heterogeneous immune cells and tumour foci in PTC. (A) Heatmap plot illustrating the prior interaction potential between ligands (immune cells) and receptors (tumour cells). (B) SpatialFeaturePlot depicting the spatial‐geneset‐score of TGFB1_Receptor_Signaling (WP) and Response_to_TGFB1 (Verrecchia) on tumour foci No. 2 and tumour foci No. 4. (C) Dot plot of the correlation of the TGFB1 pathways (TGFB1_Receptor_Signaling (WP) and Response_to_TGFB1 (Verrecchia)) and the signature genes of tumour foci No. 2 and No. 4. (D) Quantitative PCR results of the expression pattern changes of the signature genes of tumour foci No. 2 and No. 4 treated with or without TGFBR1 inhibitor (PF06952229 and AZ12601011) in TPC1 cells. (E) The count results of TPC1 cells proliferation treated with 10 μM TGFβ and/or TGFBR1 inhibitor (PF06952229 and AZ12601011). (F) Spatial‐geneset‐score expression pattern of B‐cell markers and T‐cell markers in tissue sections. (G) Clustering of immune cells into five distinct clusters in tissue sections. (H) Pie plot illustrating the percentage of the top six immune cell populations along the distribution position within the yellow area. (I) Multiplex immunohistochemical staining image showing B‐cell markers (CD20), CD8^+^ T‐cell markers (CD8) and CD4^+^ T‐cell markers (CD4) in tissue sections. (J) Statistical analysis results of the existence of TLSs in the tissue sections of PTC patients (*n* = 49, left) and the distribution of TLSs around the tumour foci by distance (*n* = 41, right). *p < .05; **p < .01; ***p < .001. (K) Violin plot displaying the expression of TGFB1 across the five immune cell clusters.

The presence and activity of immune cells within the tumour microenvironment play a significant role in shaping tumour characteristics and tumour progression. Thus, we further investigated the impact of the tumour immune microenvironment on the heterogeneity of tumour foci, with a specific focus on tumour foci No. 2 and tumour foci No. 4. To explore the interplay between immune cells and tumour cells, we performed NicheNet analysis to identify specific ligand‒receptor interactions. Our results revealed a high interaction potential between TGFβ secreted from immune cells and TGFBR1 and TGFBR2 in tumour cells (Figure 5A). Notably, the response level of TGFβ signalling was lower in tumour foci No. 2 than in tumour foci No. 4 (Figure 5B and Table S4).^59^ The TGFβ signalling pathways were positively correlated with the signature genes of tumour foci No. 4 only (Figure 5C). TGFβ signalling is known to have tumour‐suppressive effects by inducing growth arrest and apoptosis. The differential activity of TGFβ signalling in different tumour foci suggests its involvement in the outcome of PTC, as tumour foci No. 2 were associated with decreased relapse‐free survival. The results were further verified by the quantitative PCR results of TPC1 cells treated with or without TGFBR1 inhibitor (PF06952229 and AZ12601011) (Figure 5D) and cell proliferation results of TPC1 treated with TGFβ and TGFBR1 inhibitor PF and AZ (Figure 5E).

To determine the specific immune cell populations responsible for TGFβ and CXCL13 secretion within the tumour microenvironment, we analysed immune spots using multiple methods, including spatial‐geneset‐score analysis, CIBERSORT and MCPcounter analysis (Figures 5F and S7A). We identified five immune cell clusters, with clusters 1 and 3 showing high expression of B‐cell‐associated genes and cluster 3 showing high expression of T‐cell‐associated genes (Figures 5G and S7B and D). Spatial distribution analysis revealed that B‐cell‐enriched spots were primarily located at the centre, while T‐cell‐enriched spots were predominantly distributed in the periphery of the tumour (Figures 5H and S7E). Furthermore, we confirmed the presence of B cells, CD8^+^ T cells and CD4^+^ T cells (tertiary lymphoid structures [TLSs]) within typical immune areas using multiplex immunohistochemical staining in PTC tissue sections (Figures 5I and J). Additionally, we observed that TGFβ, without knowing its specific source, was distributed across all five immune cell clusters in the spatial transcriptome data (Figure 5K). We also focused on the tumour immune microenvironment surrounding the tumour foci besides TGFβ signalling. We examined the changes in features from immune cells to tumour cells. Interestingly, we observed a decrease in ligand‒receptor interactions, particularly in the CXCL13–CXCR5 and CXCL12–CXCR4 axes, along the trajectory direction (Figures S7E–S7H). These interactions are crucial for modulating the infiltration, activation and differentiation of immune cells in response to tumour antigens. Analysis of single‐cell sequencing data indicated that myeloid cells were the primary source of TGFβ production (Figure S7I).

Taken together, these findings suggest that myeloid cell‐derived TGFβ within the tumour immune microenvironment may contribute to the heterogeneity of tumour foci in PTC. The interaction between immune cells and tumour cells, particularly through ligand‒receptor pathways and the secretion of TGFβ, plays a crucial role in shaping the characteristics of different tumour foci and potentially influencing patient outcomes. Further understanding of the tumour immune microenvironment and its impact on tumour heterogeneity could have implications for the development of targeted therapies and personalised treatment strategies for PTC.

### 3.7 Validation of the cellular heterogeneity in the PTC microenvironment

**FIGURE 6:**
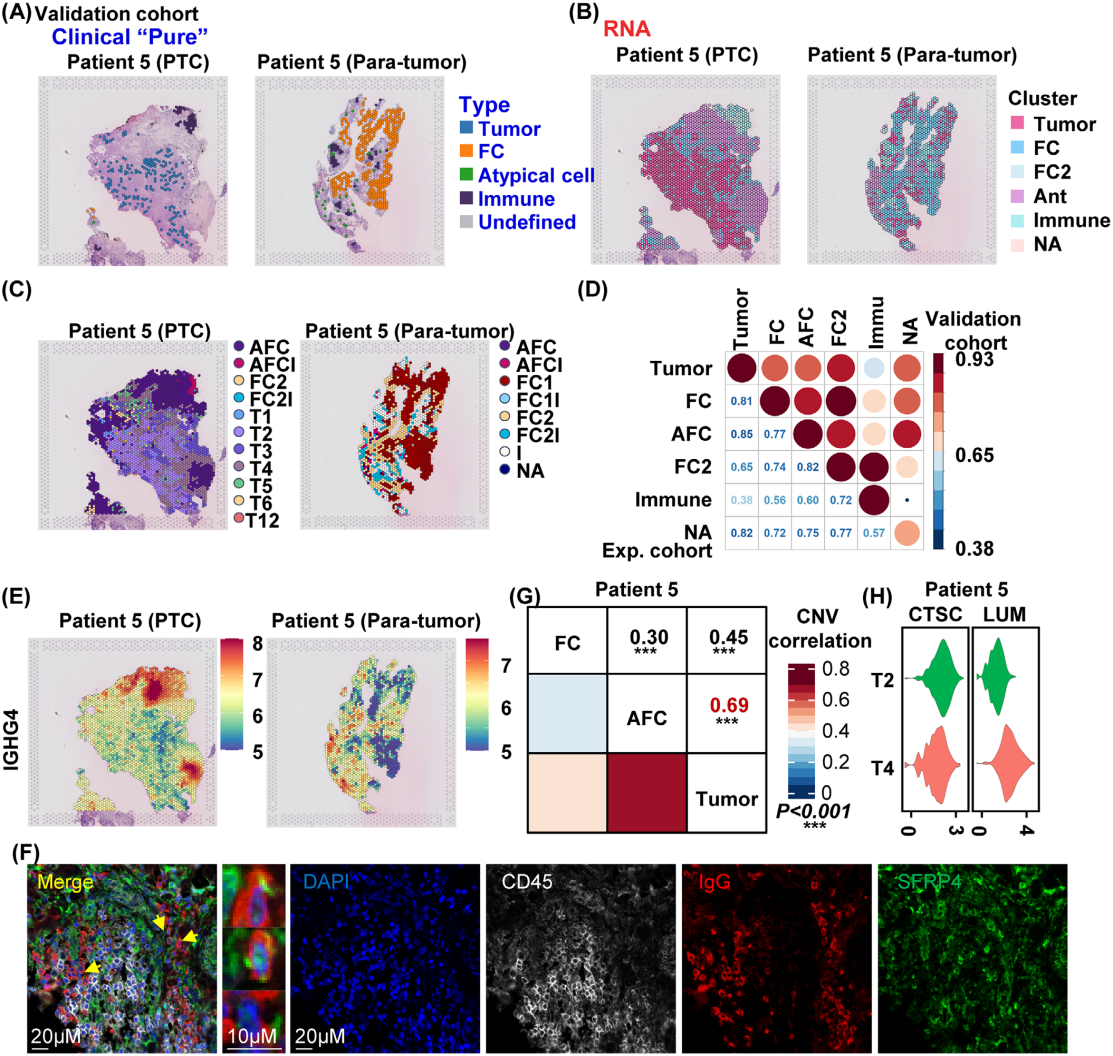
The validation cohort reveals the cellular heterogeneity in the papillary thyroid carcinoma microenvironment. (A) The corresponding clinical ‘pure’ type of PTC identified by the clinical pathologist in the validation cohort (patient No. 5). (B) Spatial mRNA identification of clusters in the validation cohort (patient No. 5) of PTC using spatial transcriptomics analysis. (C) The cell label transfer plot from patient No. 1 to other PTC samples in the validation cohort. (D) The dot plot of the Spearman correlation analysis in all PTC spatial transcriptomics samples of the experimental cohort (patient Nos. 1−4) and validation cohort (patient No. 5). (E) The spatial featureplot of IGHG4 expression in the validation cohort (patient No. 5). (F) Multiplex immunohistochemical staining image of the distribution of IgG on immune cells (CD45‐positive cells) and other cells (including AFCs and SRFP4‐positive cells) in tissue sections around tumour foci by distance. (G) The heat map of the person correlation score of CNVs in total gene across tumour, AFCs and FCs in the validation cohort (patient No. 5). FCs were used as the reference cells. (H) Violin plot of the expression of the signature genes from patient No. 1 on tumour foci No. 2 and No. 4 in the validation cohort (patient No. 5).

In addition, we added new samples in the validation cohort with PTC tumour and paratumour tissue to further proved our findings, combining histopathology and RNA expression patterns according to protocols previously described (Figures 6A, B and S8A, S8B). We also found AFCs in the cohort samples, and the common tumour foci across samples were tumour foci Nos. 2, 3 and 4 (Figure 6C). There was a high correlation score between the experimental cohort and the validation cohort, suggesting that our analytical methods are suitable for more PTC samples (Figure 6D). And the immunoglobin gene IGHG4 was highly expressed in the AFCs area (Figure 6E). The multiplex immunohistochemistry (mIHC) results on the tumour tissue of PTC also showed that IgG was also expressed on other cells besides immune cells (CD45 as a marker), including AFCs (SFRP4 as a marker) (Figure 6F). The CNV analysis also showed higher similarity of AFCs and tumour cells (Figure 6G). And the survival related genes (CTSC and LUM) isolated from tumour foci were also different in tumour foci 2 and No. 4 (Figure 6H). The distribution of immune clusters also exited in the validation cohort (Figure S8C). These data further demonstrated the heterogeneity of tumour foci, and AFCs represent a transition state from FCs to tumour cells.

## 4 DISCUSSION

Alterations in cellular heterogeneity correlate with human PTC progression and prognosis. SPT is a powerful technology for studying cellular heterogeneity. It is generally recognised that SPT is predominantly used in combination with single‐cell RNA sequencing (scRNA‐seq). However, the high cost of scRNA‐seq and the modification of the real cell state after cell suspension limit its widespread application. Moreover, the key clinical pathology information of PTC tumours provided by pathologists is usually overlooked. Pathologists are experts in distinguishing between different cells on tissues based on apparent cell morphology, differentiation status and tumour diameter. This information is considered a key determinant of clinical diagnosis. Here, we demonstrate the feasibility of a novel experimental and analytical approach to investigate the heterogeneity of PTC using SPT in combination with clinical pathologist identification. First, by combining SPT with mirror slice H&E staining, we obtained a higher resolution of the pathological structure and cell composition of PTC, enabling more accurate pathological assessments. Then, we identified RNA‐clinical signature genes through the integration of RNA clustering and clinical information and employed these genes to analyse the heterogeneity of tumour foci and the transitional state of AFCs in the progression to tumour cells. Our results uncovered the prognosis‐associated cellular heterogeneity of tumour cells, FCs and AFCs and immune cells in the PTC tumour microenvironment.

Generally, normal cells undergo a process to become transitional cells and then tumour cells. The presence of transitional cells during initial histological examination suggests the possibility of an early tumour, warranting further investigation through subsequent biopsies. Failure to identify these residual transitional cells could pose a risk. For example, low‐ and high‐grade intraepithelial neoplasia were thought to be transitional cells from normal epithelium to invasive carcinoma in oesophageal squamous‐cell carcinoma and were found to play a key role in promoting oesophageal cancer development.^60^ In addition, recent studies proposed the term ‘AFCs’ or premalignant FCs in PTC, meaning a different state from normal FCs, but it did not elucidate the tissue localisation and characteristic genes of AFCs.^8^, ^9^, ^10^, ^11^, ^12^ Here, by the spatial RNA‐clinical analysis method, our findings demonstrated that AFCs represent a transitional state between FCs and tumour cells, as evidenced by developmental trajectory analysis,^7^, ^47^ spatial distribution patterns,^44^, ^53^ characteristic gene expression levels, gene enrichment pathways and CNVs.^55^ In support of this, we also conducted a mIHC experiment and found that the number of AFCs decreased as the distance from the tumour foci increased. Our findings are consistent with scRNA‐seq data from other studies that AFCs were a transitional state between FCs and tumour cells, exhibiting a higher resemblance to the latter.^4^, ^22^ To facilitate clinical translation, the specific diagnostic molecules associated with AFCs should be identified and validated in future studies. How AFC ultimately affects the outcome of human PTC is also worth investigating.

Recent insights have shown that the heterogeneity of tumour foci can significantly affect tumour prognosis. For example, distinct tumour foci exist in primary prostate cancers, and sequencing studies have shown that tumour foci heterogeneity poses obstacles for the diagnosis and treatment of prostate cancer.^61^ In contrast to some studies that used only scRNA‐seq, our study focused on both the tumour cell level and the tissue and distribution heterogeneity of tumour foci. In our study, we successfully explored the heterogeneity of PTC tumour foci through signature gene extraction, pathway enrichment analysis, evolutionary state analysis and survival analysis.^52^ Three tumour foci were found to be shared among all patients out of the 13 observed, and these foci were focused on in the study of the relationship with PTC prognosis. Notably, tumour foci No. 2 displayed elevated expression levels of genes associated with lower relapse‐free survival in PTC patients. Furthermore, our ContourPlot analysis model enabled the exploration of ligand‒receptor interactions within tumour cells, AFCs and FCs.^54^ We identified several enriched pathways, including FN1–ITGA3, LAMB3–ITGA2 and FN1–SDC4, along the trajectory from tumour to AFC to fully transformed cells. Moreover, high expression of these ligand receptors was associated with decreased relapse‐free survival in PTC patients. These interactions also exist in other cancers, such as pancreatic adenocarcinoma.^62^ However, here, their role in PTC has been proposed for the first time. Understanding how the tumour microenvironment shapes tumour cells and their distinct functional properties within different tumour foci, including ligand‒receptor interactions, distances from normal tissues, immune microenvironment and gene expression patterns, will be crucial for future investigations. Further investigation is warranted to elucidate the detailed mechanisms by which these ligand‒receptor interactions benefit tumour cells.

Multiple health outcomes are associated with the types and status of immune cells in the tumour microenvironment. For example, TLSs, including T and B cells within and outside the tumour, have different prognostic impacts on patients with intrahepatic cholangiocarcinoma.^63^, ^64^ In our study, reduced interaction between myeloid‐derived TGFB1 and TGFBR1 in tumour foci No. 2 contributed to tumourigenesis and increased heterogeneity, which was support by the spatial‐geneset‐score and nichenet analysis, quantitate PCR and cell proliferation validation results. Additionally, we observed alterations in the CXCL13–CXCR5 and CXCL12–CXCR4 ligand‒receptor interactions between immune cells and tumour cells, which potentially play important roles in modulating the infiltration, activation and differentiation of immune cells in response to tumour antigens.^65^, ^66^, ^67^ These findings reveal potential molecules and methods by which immune cells remodel tumour foci heterogeneity and thus affect the prognosis of PTC patients. Extensive clinical trials are needed to further verify the key effects of TGFB1 or CXCL13–CXCR5 and CXCL12–CXCR4 axes in PTC treatment.

One limitation of the 10× Visium SPT protocol is its low resolution, which can lead to suboptimal staining effects and affect sequencing quality. To overcome this limitation, we improved the imaging results of H&E staining on mirror slides, enabling pathologists to clearly define pathological areas. By combining the advantages of clinically identified area types with high‐throughput gene expression data, we obtained pure RNA‐clinical cells and signature genes, which were integrated with spatial transcriptome data. This approach allowed for spatial‐geneset‐score analysis, precise cell identification and comprehensive clinical analysis of the tumour microenvironment. Another limitation in this study is that pathologists did not find the typical cancer‐associated fibroblasts (CAFs) area in patient No. 1, which is important in the PTC tumour microenvironment, so we did not further investigate the relationship between FCs, tumour cells and CAFs. The heterogeneity of thyrocytes in PTC is well documented.^4^, ^6^, ^7^ Our investigation identified a distinct cluster of follicular cells, denoted as FC2, characterised by the expression of immunoglobulin genes, normal nuclei morphology identified by the clinical pathologist and typical FC gene expression (e.g., TFF3 and TPO). Different from FC2, the expression pattern of AFCs demonstrated greater similarity to tumour cells than FC cells. And it is particularly intriguing that the AFC spots highly express many immunoglobulin genes, including IGHG4. Recent findings have demonstrated that tumour cells and tissue cells can express immunoglobulin genes.^68^, ^69^, ^70^, ^71^, ^72^, ^73^ Therefore, as the transitional states of the tumour cells, AFC cells may also express high‐level IGHG4‐related IgG. So, we mainly focused on the immunoglobulin genes expression in AFCs. Both the mIHC data and the validation cohort data further proved that immune‐related genes (IGHG4) were highly expressed in AFC cells, especially in tumour tissue compared with tumour adjacent tissue. However, the functional implications of immunoglobulin expression in AFCs within the context of PTC remain uncertain.

**FIGURE 7:**
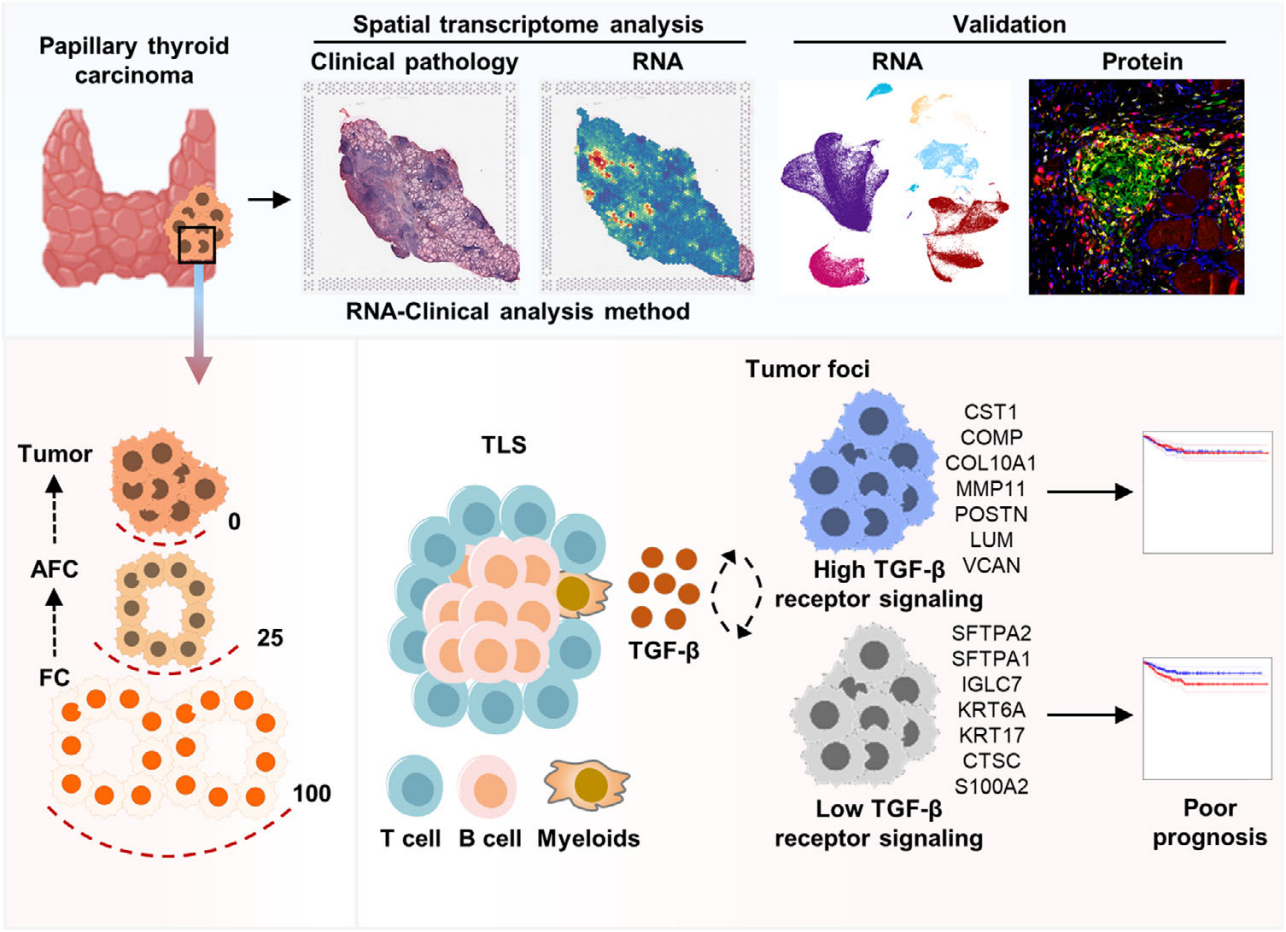
Schematic representation of the spatial transcriptomics reveals prognosis‐associated cellular heterogeneity in the papillary thyroid carcinoma microenvironment.

In summary, our study introduced a novel approach to examine the heterogeneity of PTC using SPT and mirror slice H&E staining. We demonstrated the utility of this approach in integrating pathological information with gene expression data, enabling comprehensive analyses of the tumour microenvironment and identification of clinically relevant features. Our findings elucidated the transitional state of AFCs, the role of immunoglobulin expression in PTC, and the importance of ligand‒receptor interactions in tumour progression and immune responses (Figure 7). By uncovering the heterogeneity of tumour foci, this study contributes to a more comprehensive understanding of PTC biology and provides valuable insights for future diagnostic and therapeutic strategies.

## AUTHOR CONTRIBUTIONS

*Conceptualisation*: K. Y. and B. X. *Methodology*: K. Y., Q. Z. L. and R. R. H. *Software*: K. Y., Y. H. J. and Z. H. B. *Validation*: K. Y. and R. R. H. *Formal analysis*: K. Y. and Q. Z. L. *Investigation*: K. Y. *Resources*: K. Y. and S. J. L. *Data curation*: K. Y. *Writing – original draft preparation*: K. Y., R. R. H. and Y. H. *Writing – review and editing*: Z. X. L., Z. B. Z., L. L., Q. Z. L., Q. L. Z., H. X. G., R. R. H. and K. T. *Visualisation*: H. B. and Y. H. J. *Supervision*: Z. X. L., H. X. G. and B. X. *Project administration*: Z. X. L. and B. X. *Funding acquisition*: Z. X. L., H. X. G. and K. Y.

## CONFLICT OF INTEREST STATEMENT

The authors declare that the research was conducted in the absence of any commercial or financial relationships that could be construed as potential conflicts of interest.

## ETHICS STATEMENT

All procedures and the use of tissue were performed according to recent national ethical guidelines. This study was conducted in accordance with the recommendations of the Guide for the Medical Ethics Committee of the Second Affiliated Hospital of the South China University of Technology (K‐2019‐185). Written informed consent was obtained from all participants included in the study.

## Supporting information

Supporting Information

Supporting Information

Supporting Information

Supporting Information

Supporting Information

Supporting Information

## Data Availability

Raw sequencing data have been deposited in the Genome Sequence Archive (GSA‐human: HRA003537) and are accessible at https://ngdc.cncb.ac.cn/gsa‐human/s/O0X38Rcb. The major steps for processing the spatial transcriptome were integrated as an R package in GitHub (https://github.com/AlexyanKai/Spatial‐pathology‐pipeline). Supplementary Table 5 provides a summary of the source of the materials, instruments and analysis tools. All study data are included in the article and/or supplementary material.
