# Supplementary material for: Spatial transcriptomics reveals prognosis‐associated cellular heterogeneity in the papillary thyroid carcinoma microenvironment
Source: Clin Transl Med. 2024 Mar 1;14(3):e1594. doi: 10.1002/ctm2.1594 (PMC10905537; doi:10.1002/ctm2.1594)
Supplement: Supplementary file 1 — Supporting Information [file CTM2-14-e1594-s006.docx]

Supplementary Figures

**Spatial transcriptomics reveals prognosis-associated cellular heterogeneity in the papillary thyroid carcinoma microenvironment**

Kai Yan^1, #^; Qing-Zhi Liu^2, #^; Rong-Rong Huang^1, #^; Yi-Hua Jiang^1,3^; Zhen-Hua Bian^4^; Si-Jin Li^5^; Liang Li^6^; Fei Shen^5^; Koichi Tsuneyama^7^; Qing-Ling Zhang^8, *^; Zhe-Xiong Lian^9^; Haixia Guan^10, *^; Bo Xu^5, *^

Supplementary Figure legend


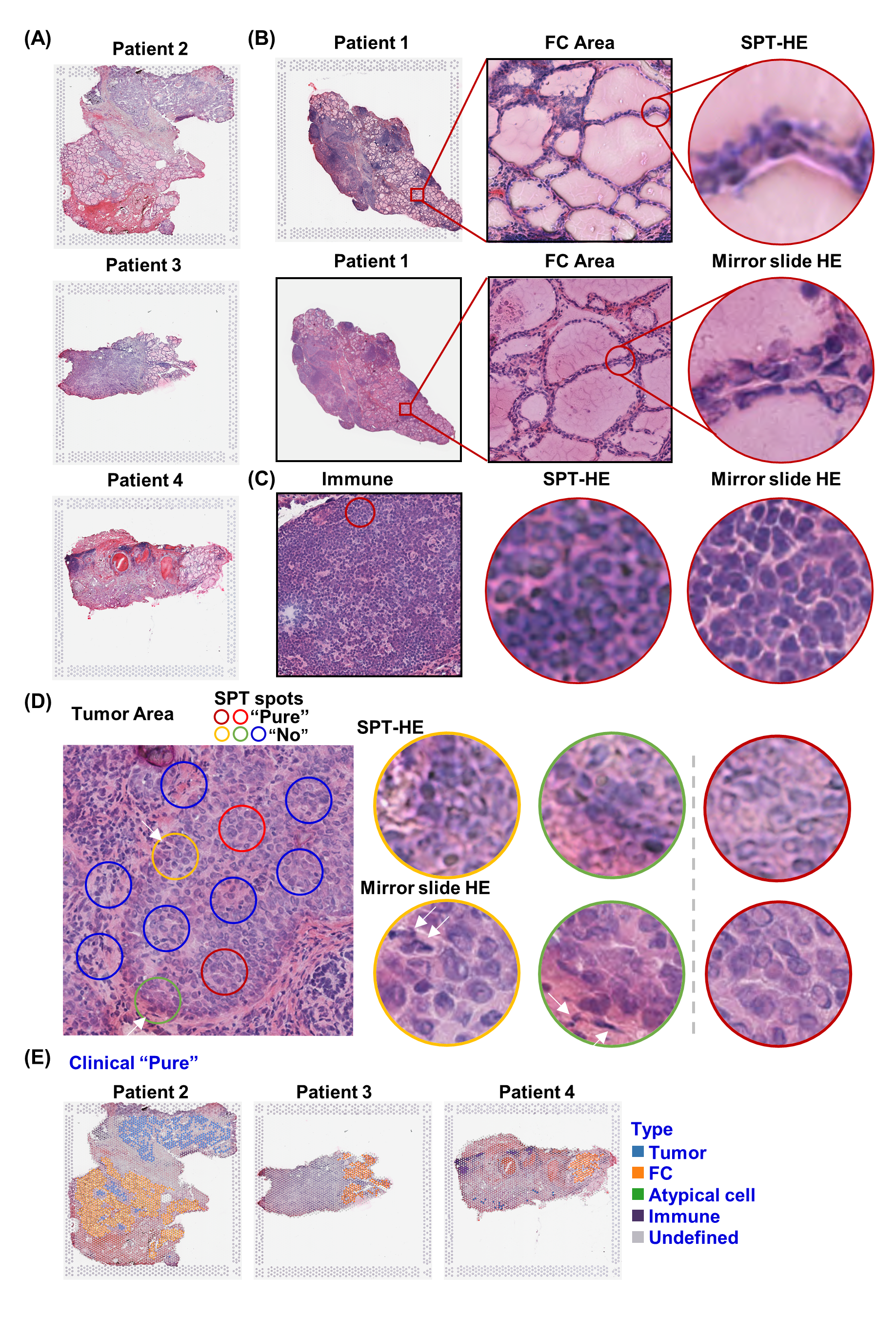


**Figure S1. Improved HE staining image on spatial slides and mirror slides of papillary thyroid carcinoma.** **(A)** HE staining results on spatial transcriptomics slides (SPT-HE) from all four PTC patients. And magnification images of selected FCs area on spatial transcriptomics slide. **(B)** Improved HE staining (HE) results and magnification images of FCs area on mirror slide of papillary thyroid carcinoma spatial transcriptomics slide. **(C)** Magnification images of improved HE staining (HE) and spatial HE staining (SPT-HE) results on selective Immune area of PTC. The clinical pathologist accurately identified the “Pure” immune spot (red cycle) in the typical immune area. **(D)** Magnification images of improved HE staining (HE) and spatial HE staining (SPT-HE) results on selective Tumor area of PTC. The clinical pathologist accurately identified the “Pure” tumor spot (red cycles) in the typical Tumor area and “no pure” tumor spot (blue, green and yellow cycles). White arrows marked the cells with noticeably different nuclei staining from the surrounding tumor cells. **(E)** The Clinical “Pure” type of papillary thyroid carcinoma identified by clinical pathologist in patient No. 2, 3 and 4.


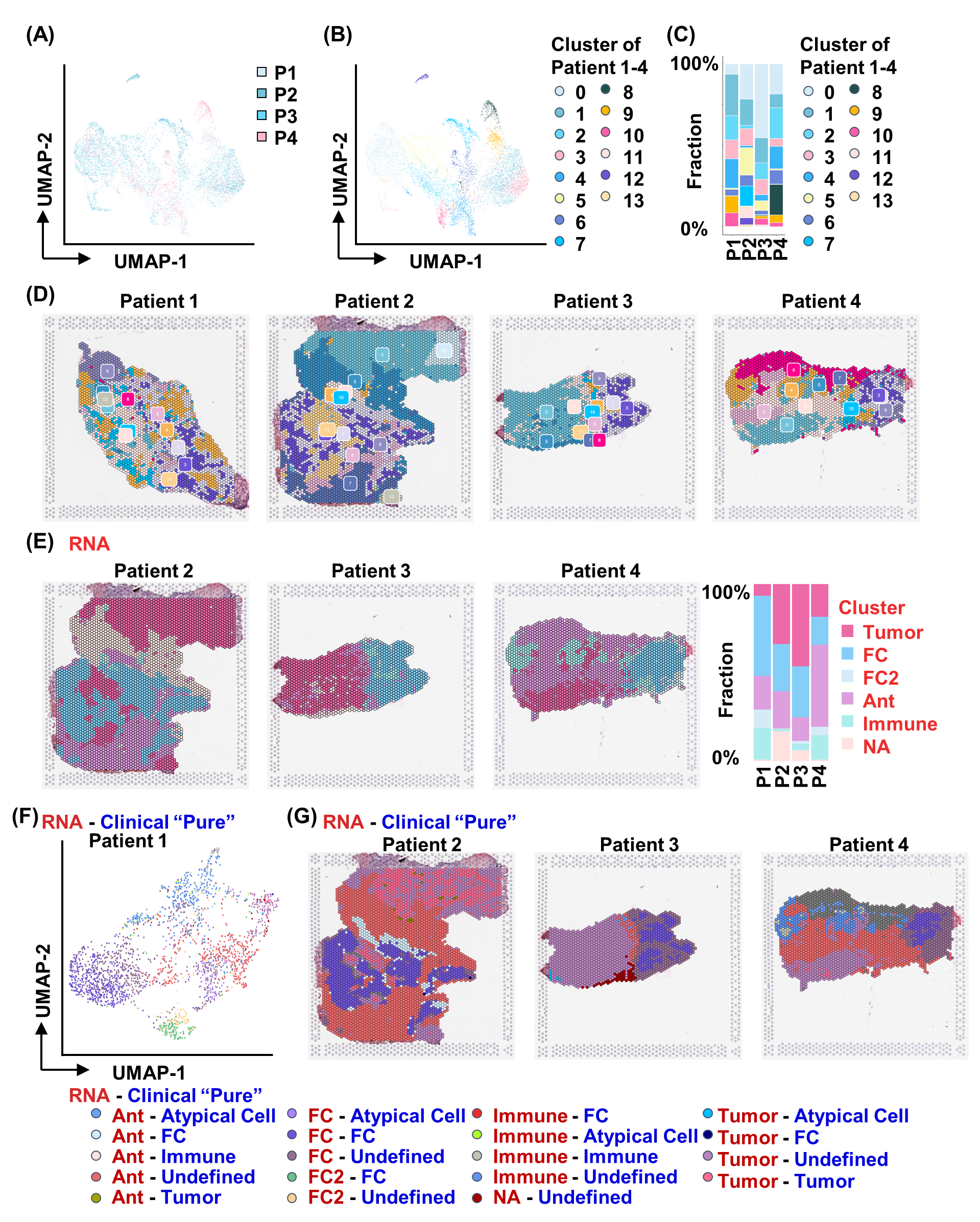


**Figure S2. RNA analysis results of PTC spatial transcriptomics.** **(A)** UMAP (Uniform manifold approximation and projection) plot of tissues from all sections. **(B)** UMAP clusters plot of merged spatial transcriptomics from all patients, colored by their clusters ID. UMAP were performed by the first 20 principal components (PCs). **(C-D)** Spatial distribution of clusters on all patients (D). The fraction of clusters from all patients (C). **(E)** Spatial distribution of RNA identified clusters (Tumor, FCs, Immune, Ant, FC2 and NA) on all patients (left). The fraction of RNA identified clusters (Tumor, FCs, Immune, Ant and NA) on all patients. Tumor (tumor cells), FCs (follicular cells), Immune, Ant (adjacent non-cancerous tissue, including fibroblasts and blood vessel cells) and NA (unidentified cells) were identified from the 14 clusters of merged spatial transcriptomics of PTC (right). **(F)** UMAP clusters plot of spatial transcriptomics from patients A, colored by RNA cluster (red letters) and clinical type (blue letters). (**G**) The merged analysis image of patients Nos. 2, 3 and 4 displays the RNA clusters (indicated by red letters) and clinical types (indicated by blue letters).


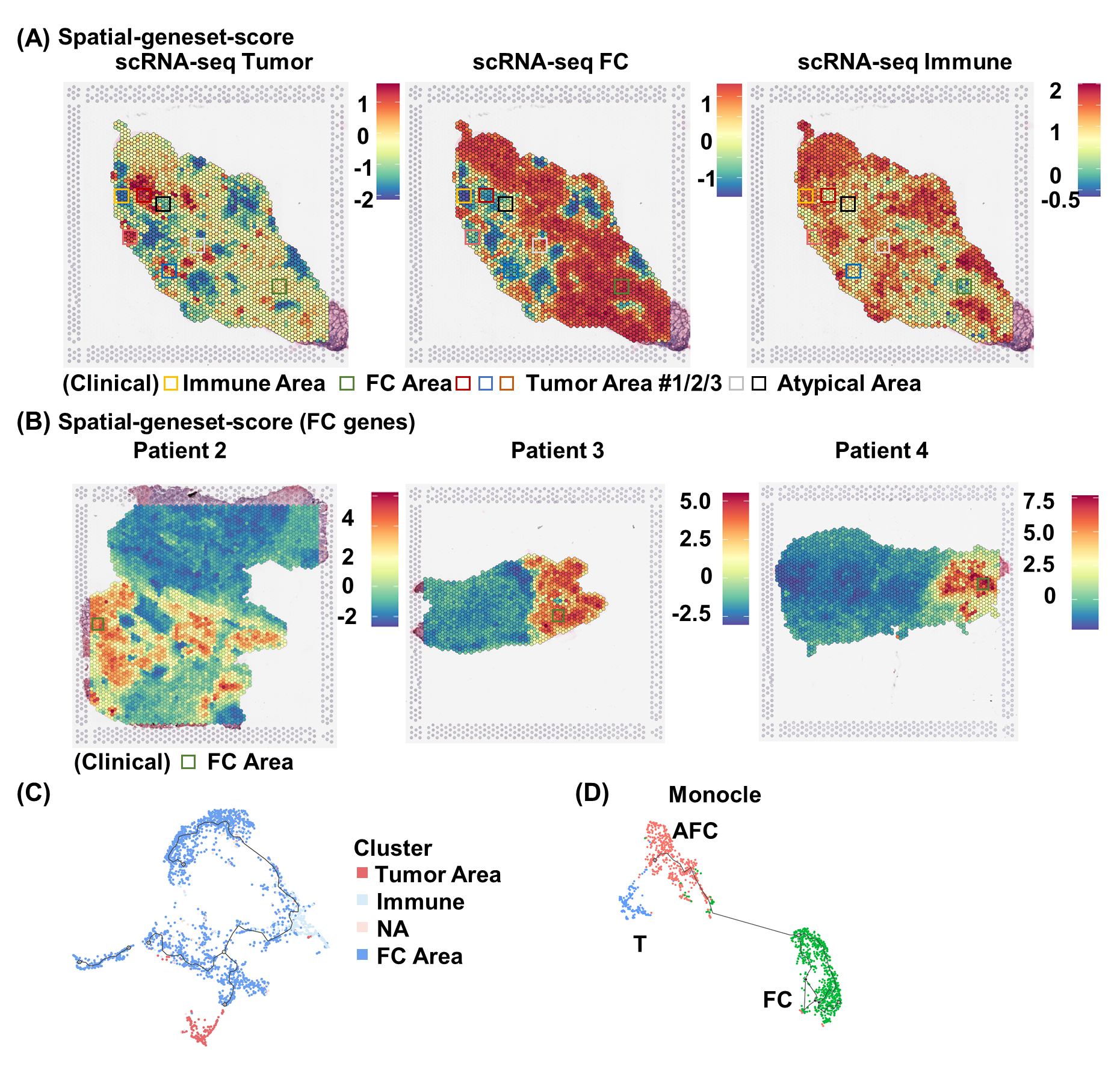


**Figure S3. The application of signatures of RNA-clinical cluster and spatial-geneset-score.** **(A)** Expression patterns of tumor markers, FCs markers, and immune markers isolated from scRNA-sequencing data in tissue sections from patient No.1. **(B)** Expression patterns of FCs markers in tissue sections from all patients. **(C)** The pseudotime plot of Immune, Tumor area, FCs area and NA in tissue sections calculated by Monocle 3 (in the SPATA2). **(D)** The pseudotime plot of Tumor, FCs area and AFCs in tissue sections. Tumor, FCs area and AFCs were selected and recalculated for the developmental state analysis.


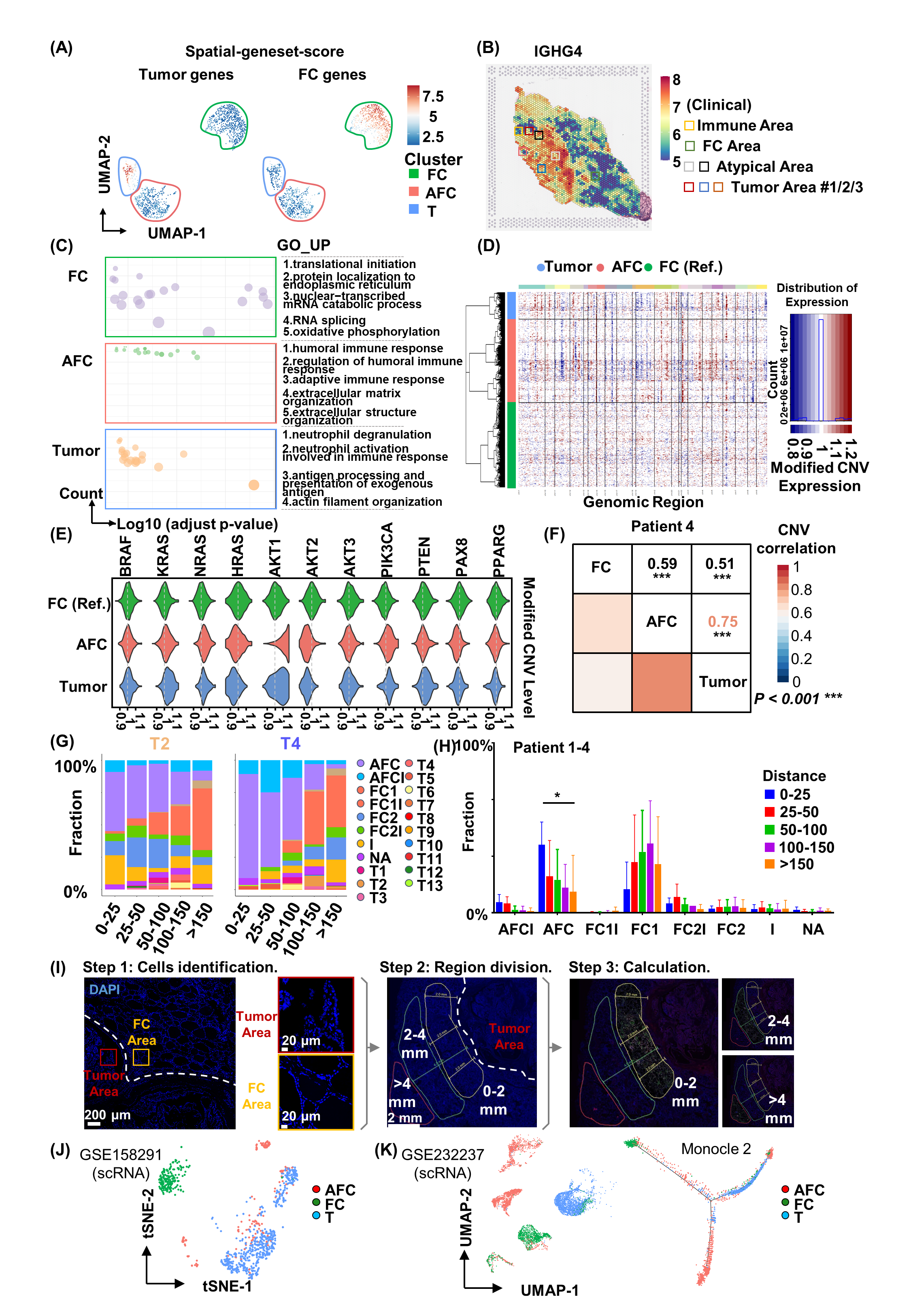


**Figure S4. The atypical follicular cells were transition cells from follicular cells to tumor cells. (A)** The feature plot of Tumor (left) and FCs (right) signature genes on Tumor, AFCs and FCs cells based on spatial-geneset-score method. **(B)** The spatial featureplot of IGHG4 in patient No.1. **(C)** Up-regulated GO pathway in Tumor, AFCs or FCs cells compared with other cells. (**D**) The CNV expression of FCs, AFCs and Tumor were calculated by inferCNV. FCs were used as the reference group. The distribution of CNV expression (left) and the clustering analysis result of the cells performed on the overall CNV levels (middle) were showed. The heatmap plot of modified CNVs expression in Tumor (blue bar), AFCs (red bar), and FCs (green bar) across the genomic region (right). Horizontal lines represent individual cells, and vertical lines represent genes in the heatmap. (**E**) The violin plot of modified CNVs expression of BRAF, RAS, AKT, PIK3CA, PTEN, PAX8 and PPARG in Tumor (blue bar), AFCs (red bar) and FCs (green bar). FCs (FC=1) were used as the reference group, which means that the modified CNVs expression of overall genes in FCs were 1.0. (**F**) The heatmap of the person correlation score of CNVs in total gene across Tumor, AFCs, and FCs in patient No. 4. FCs were used as the reference cells. **(G)** Bar chart of cell fraction of external microenvironment around tumor foci 2 and tumor foci 4 grouped by distances in patient No.1. **(H)** The statistical analysis bar chart of cell fraction of tumor foci external microenvironment grouped by distances in all samples. (**I**) The process of how tumor foci were identified and the distance of atypical follicular cells from tumor foci calculated were detailed descript below. Step 1: the reorganization of typical Tumor area and FC area based on DAPI staining; Step 2: delineate areas at distances of 2 mm and 4 mm from typical tumor regions in HALO software; Step 3: calculate the percentage of SFRP4^+^ cells in DAPI^+^ cells in 0-2 mm, 2-4 mm and >4 mm area around the typical tumor area. **(J)** tSNE plot illustrating the distribution of Tumor, AFCs and FCs cells in the single-cell sequencing data (GSE158291) across the PTC patients. **(K)** tSNE plot illustrating the distribution of Tumor, AFCs and FCs cells in the single-cell sequencing data (GSE232237) across the PTC patients (left). Monocle plot illustrating the development state of Tumor, AFCs and FCs cells in the single-cell sequencing data (GSE232237) (right).


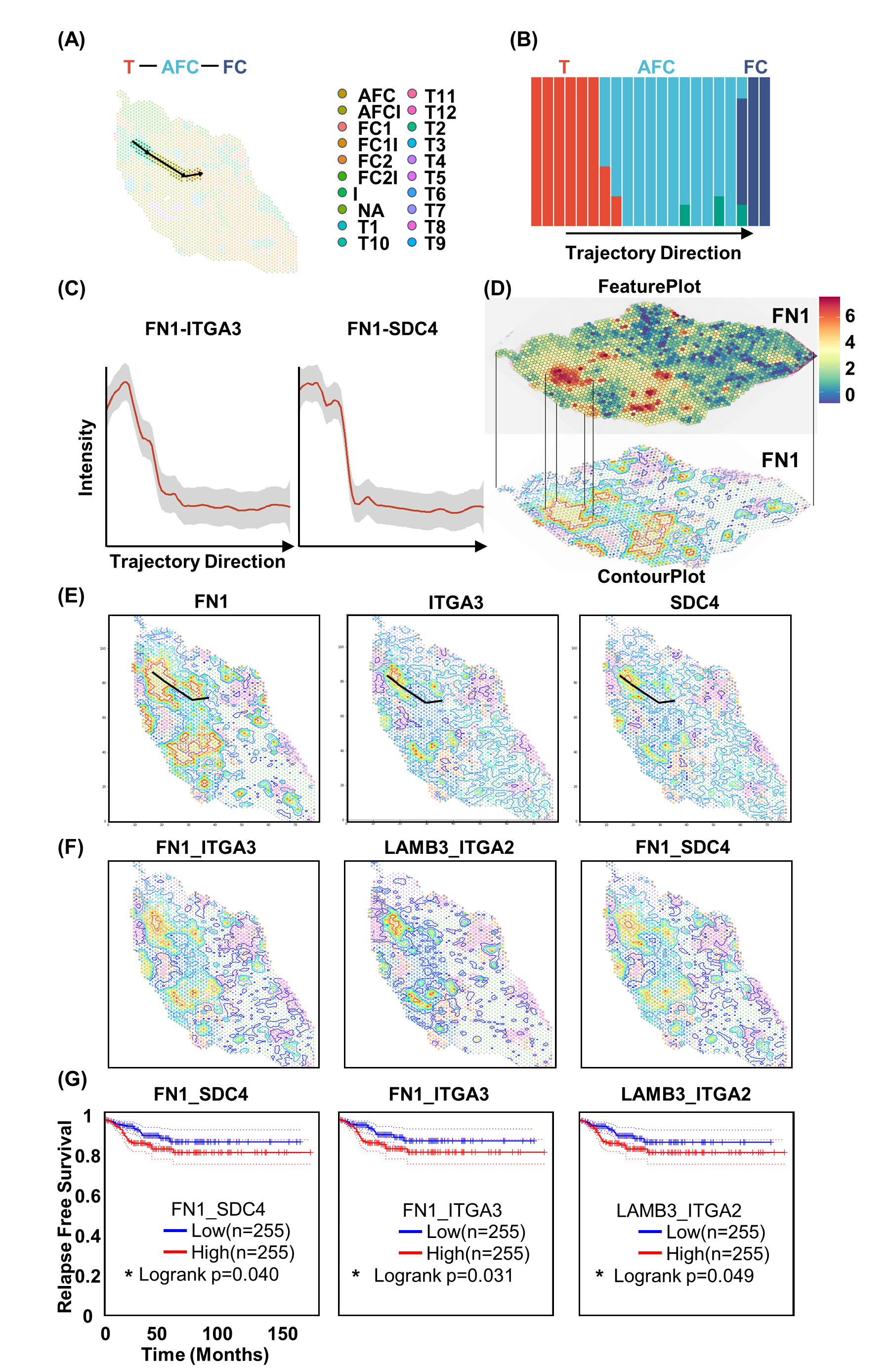


**Figure S5. The typical ligand-receptor changes of PTC and ContourPlot analysis.** **(A-B)** The trajectory segmentation of tumor external microenvironment (**A**) and cell fraction along the trajectory (**B**). **(C)** The FN1-ITGA3 and FN1-SDC4 ligand-receptor interaction intensity along the trajectory of tumor external microenvironment. **(D)** The schematic diagram of FN1 ContourPlot converted from FeaturePlot. **(E)** The contour plot of FN1, ITGA3, SDC4 in PTC tissues along the trajectory. **(F)** The contour plots of top 3 enriched ligand-receptor pathway FN1-ITGA3, LAMB3-ITGA2, and FN1-SDC4 in tumor microenvironment. **(G)** The relapse free survival plot of THCA (TCGA database) by signature genes from top 3 enriched ligand-receptor pathway.


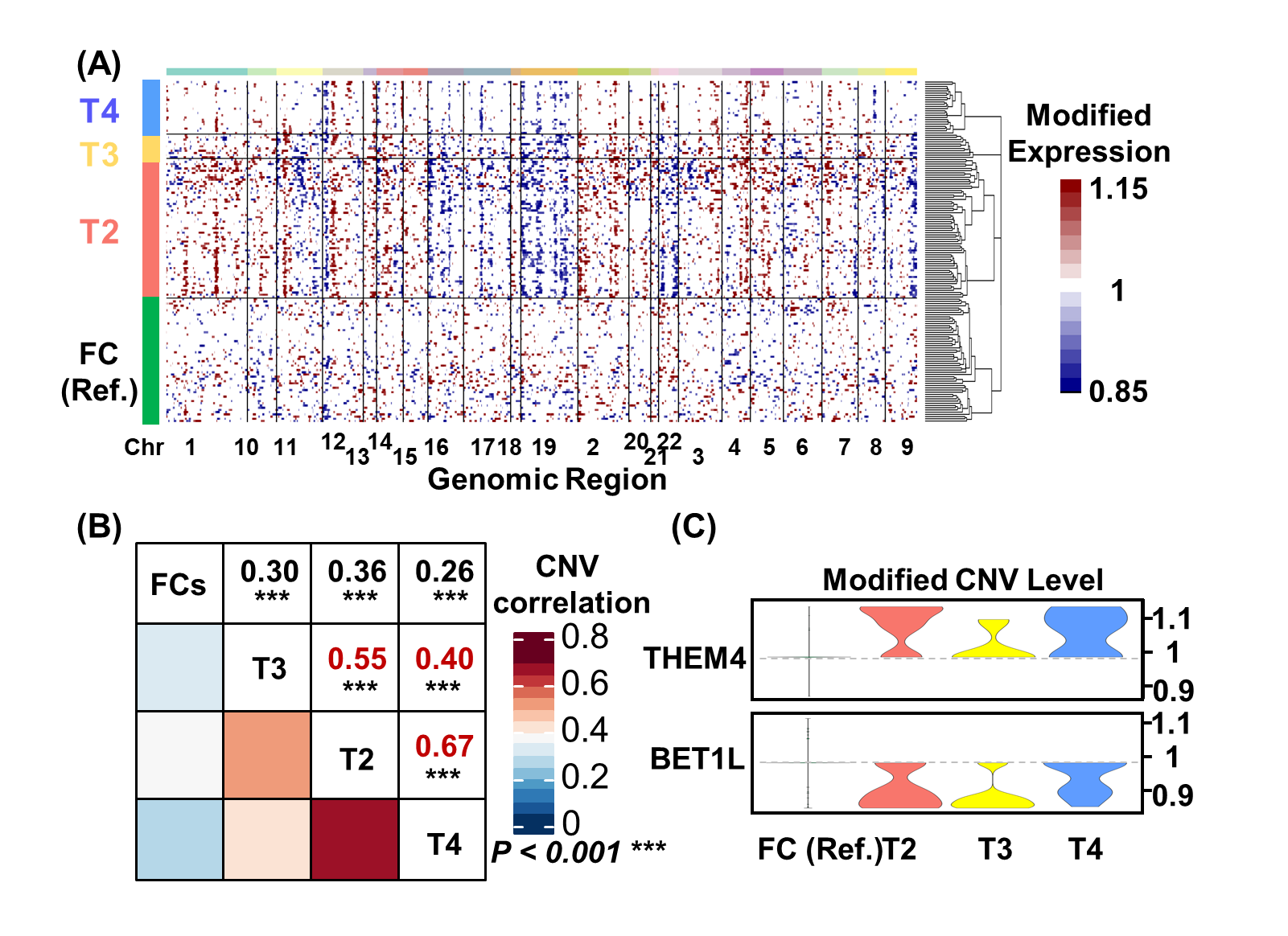


**Figure S6. The heterogeneity of tumor foci. (A)** The heatmap plot of modified CNVs expression in tumor foci No. 2 (red bar), tumor foci No.3 (yellow bar), tumor foci 4 (blue bar), and FCs (green bar) across the genomic region in patient 1. FCs were used as the reference group. **(B)** The heat map of the person correlation score of CNVs in total gene across tumor foci No. 2, 3 and 4 in patient No. 1. **(C)** Violin plot of modified CNV level on the top gain (THEM4) and loss (BET1L) mutation genes across tumor foci No.2, tumor foci No.3, tumor foci No.4 and FCs. FCs were used as the reference cells.


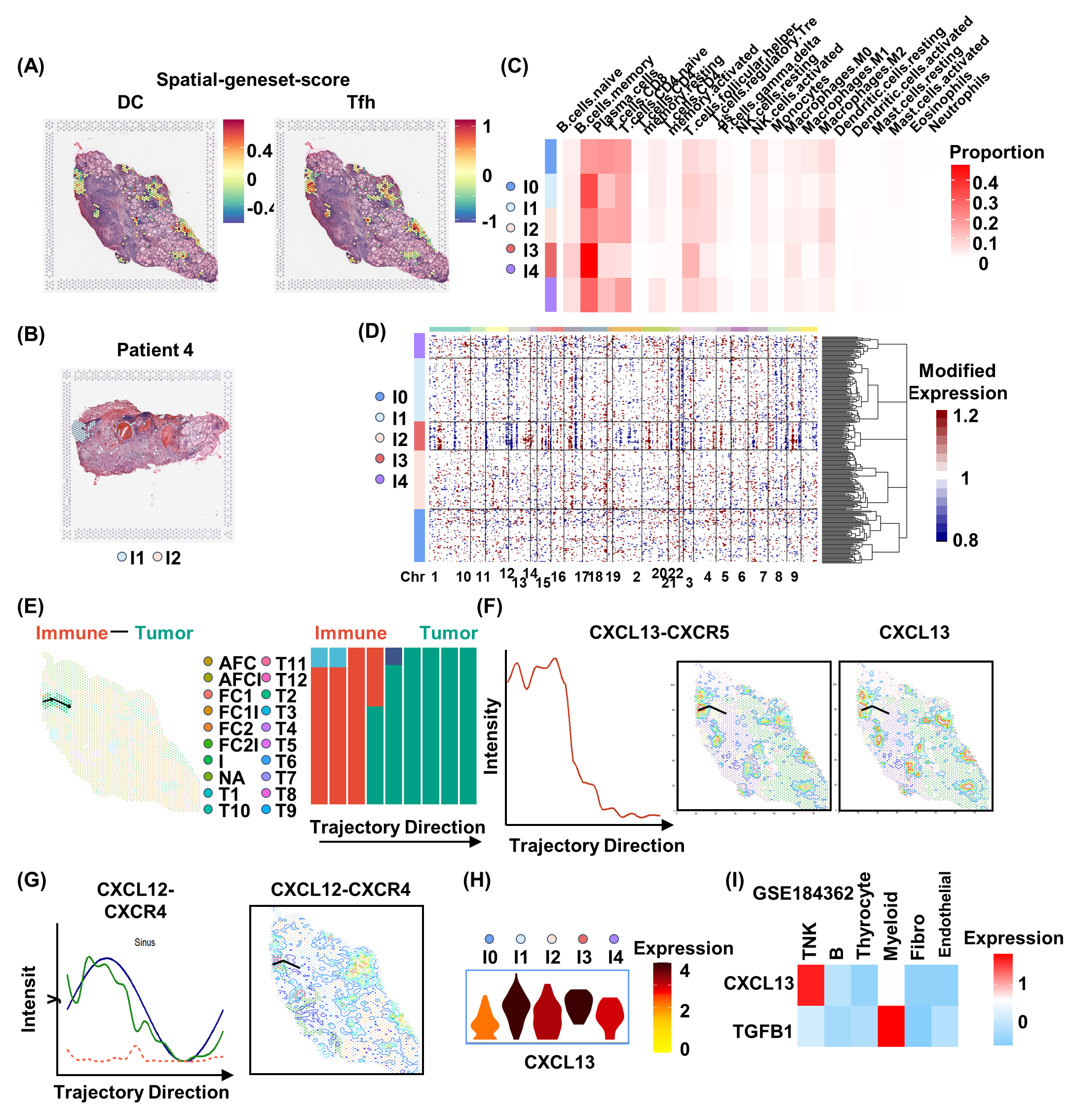


**Figure S7. The immune-tumor interaction of PTC.** **(A)** The spatial-geneset-score expression pattern of DC cell markers, and Tfh cell markers in tissue sections. **(B)** The distribution of immune cell cluster across samples (patient No. 4). **(C)** Heatmap of the expression distribution score (calculated by Cibersort) of subpopulation lymphocytes on the 5 cluster of immune cells. **(D)** The heatmap plot of modified CNVs expression in the 5 clusters of immune cells across the genomic region. The cluster 0 of Immune (I0) were used as the reference group. **(E)** Trajectory segmentation from immune cells to the tumor area (left) and cell fraction distribution along the trajectory (right). **(F)** Intensity of the CXCL13-CXCR5 ligand-receptor interaction along the trajectory of the tumor's external microenvironment (left). Contour plots displaying the enrichment of the CXCL13-CXCR5 ligand-receptor pathway in the tumor microenvironment (right). **(G)** The CXCL12-CXCR4 ligand-receptor interaction intensity along the trajectory of tumor external microenvironment (left). The contour plots of CXCL12-CXCR4 enriched ligand-receptor pathway in tumor microenvironment (Right). **(H)** Violin plot of CXCL13 expression across the five cluster of immune cells. (**I**) UMAP plot illustrating the distribution of clusters in the single-cell sequencing data (left, GSE184362) and heatmap depicting the expression of TGFB1 and CXCL13 across the PTC clusters (right).


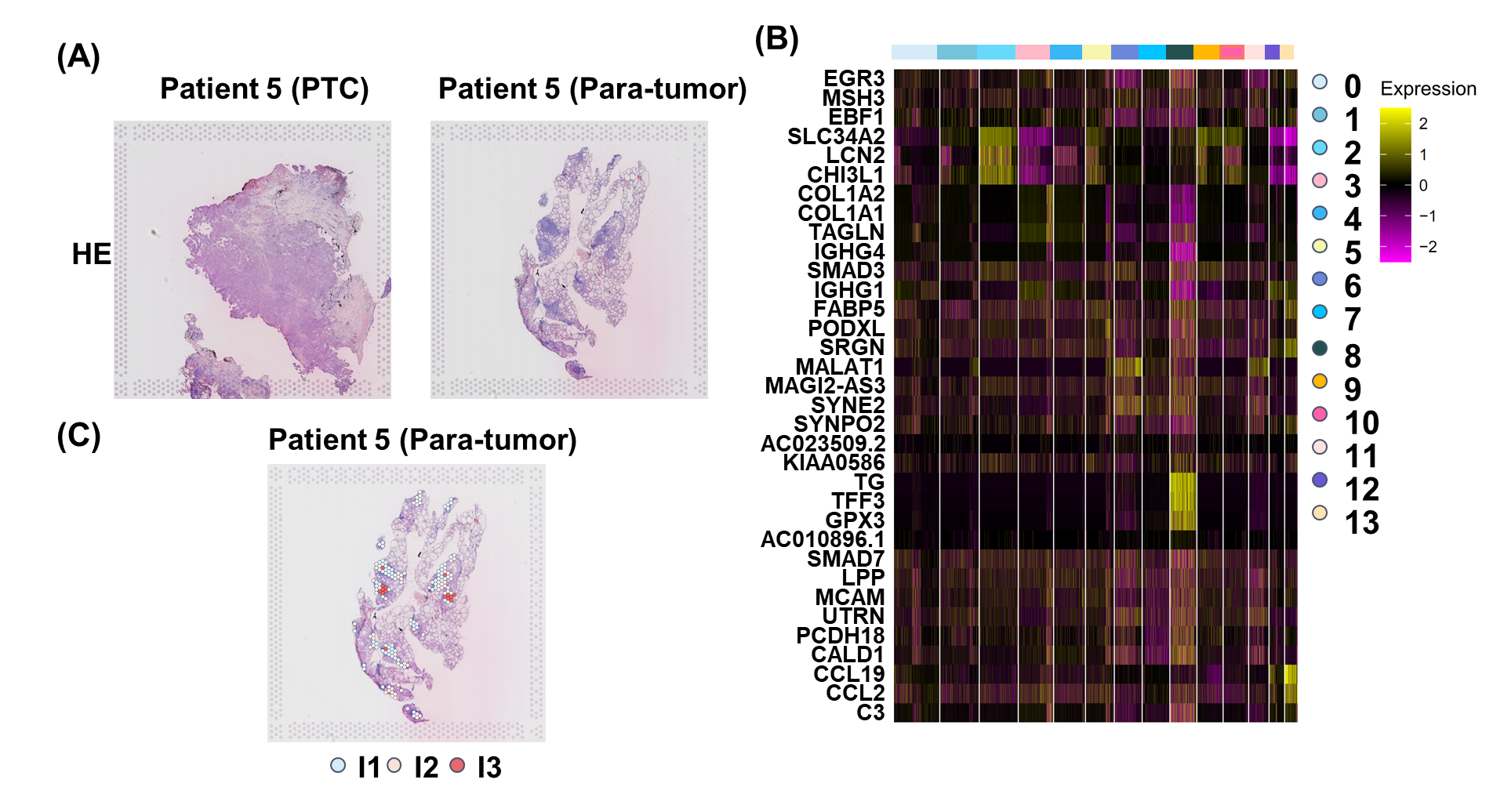


**Figure S8. The validation cohort of PTC. (A)** HE staining results on spatial transcriptomics slides (SPT-HE) from all PTC patients in the validation cohort (Patient No.5). **(B)** Heatmap plot of feature genes expression pattern across the 14 clusters on merged spatial transcriptomics of PTC. **(C)** The distribution of immune cell cluster across the validation samples (patient No. 5).
